# Burden of hypertensive heart disease among women of childbearing age in global, regional, and national regions from 1990 to 2021 and projection until 2040

**DOI:** 10.3389/fgwh.2025.1600340

**Published:** 2025-07-11

**Authors:** Zhenglong Wang, Hao Li, Hongwei Wei, Baotang Liu, Lei Li, Zijun Li, Jindong Feng, Yunjie Hu, Xiaobo Liu

**Affiliations:** ^1^ School of Clinical Medicine, Shandong Second Medical University, Weifang, China; ^2^ Department of Cardiovascular Surgery, Affiliated Hospital of Shandong Second Medical University, Weifang, China; ^3^ Department of Gynecology, Affiliated Hospital of Qingdao University, Qingdao, China

**Keywords:** hypertensive heart disease, global burden of disease, women of childbearing age, cardiology, public health

## Abstract

**Background:**

The present research focuses on elucidating the global burden of hypertensive heart disease (HHD) among women of childbearing age (WCBA). By utilizing all available data and adopting the methodology employed in the Global Burden of Disease (GBD) study, this study aims to comprehensively analyze the epidemiological characteristics of this highly prevalent disorder.

**Methods:**

In this research, we retrieved three key indicators—prevalence, incidence, and DALYs—related to HHD in WCBA from the GBD database for the period 1990 to 2021. Our study provides point estimates along with their corresponding 95% uncertainty intervals (UIs). The evolving trends were assessed using the EAPC and percentage change.

**Results:**

In 2021, the global prevalence of HHD among WCBA was 544,544 cases, resulting in 25,669 deaths. Compared to 1990, the prevalence increased by 110%, and mortality rose by 27%. From 1990 to 2021, the prevalence rate showed an upward trend (EAPC: 1.31), whereas the mortality rate exhibited a decline (EAPC: −0.51). In 2021, middle SDI regions recorded the highest prevalence rate (30.88 per 100,000), while low SDI regions had the highest mortality rate (2.44 per 100,000) and the greatest burden of DALYs (125.39 per 100,000). Among different age groups in 2021, the 45–49 age group experienced the highest number of cases (195,288) and the highest prevalence rate (82.87 per 100,000). Furthermore, HHD prevalence demonstrated a positive correlation with age.

**Conclusion:**

Overall, on a global scale, the burden of HHD among WCBA has significantly increased over the past 32 years. This trend is particularly pronounced in low SDI regions and within the 45–49 age group. The findings of this study highlight the critical need for targeted interventions to address this issue.

## Introduction

Hypertension, defined as a systolic/diastolic blood pressure of 140/90 mmHg or higher, constitutes a major risk factor for cardiovascular diseases (CVD). It is recognized globally as the leading cause of DALYs (1–3). HHD arises from prolonged pressure and volume overload in hypertensive patients. This overload increases afterload, myocardial wall stress, and stimulates fibroblast proliferation, ultimately leading to left ventricular hypertrophy (LVH), which serves as the primary pathological hallmark of HHD (4, 5). LVH develops as a result of sustained high blood pressure over extended periods and is associated with various clinical manifestations, such as myocardial thickening, arteriosclerosis, and cardiac arrhythmias (6). As a key complication of hypertension, HHD significantly contributes to the growing global burden of cardiovascular disease (7–9).

Hypertension remains a significant health concern for women (10, 11). It is a critical modifiable risk factor for CVD, which is a leading cause of mortality among women (12). Hypertension adversely affects quality of life (13). Over the past few decades, considerable efforts have been directed toward identifying and improving women's health, particularly in the areas of preventing and managing health issues specific to women (14–17). A review of over 1.5 million medical research articles indicates that including more women in research studies correlates with an increased emphasis on gender- and sex-specific factors within disease-focused investigations (18).

Consequently, it is imperative to provide a comprehensive depiction and evaluation of the overall disease status and evolving trends of HHD among WCBA. By utilizing the latest data from the GBD 2021 study, an in-depth analysis of prevalence, mortality, and DALYs was conducted at global, regional, and national levels spanning from 1990 to 2021. This analysis specifically focused on comparing the distribution and changes in the burden of HHD across different ages, time periods, and geographical regions.

## Method

### Data source

GBD estimates the disease burden for 371 diseases and injuries and 88 risk factors across 204 countries and territories. Additional details regarding the location, disease, and risk hierarchies utilized in GBD 2021 can be accessed via the Global Health Data Exchange(GHDx) (19, 20), with comprehensive information on data sources, methodologies, and statistical modeling provided in prior reports (21). This study utilizes the most recent epidemiological data and employs advanced, standardized methodologies for its assessments (22). The GBD 2021 study estimated incidence, prevalence, and mortality rates, as well as years of life lost (YLLs), years lived with disability (YLDs), and disability-adjusted life years (DALYs). DALYs is a standard metric for quantifying burden, represent the total years of healthy life lost from the onset of a disease to death, encompassing both YLLs and YLDs, as expressed by the formula: DALYs = YLLs + YLDs. In this study, we present the numbers and rates of prevalence, mortality, and DALYs associated with HHD in 2021, along with their temporal trends from 1990 to 2021. These estimates were further stratified by age group, time period, and geographical region.

### Socio-demographic index (SDI)

The Socio-demographic Index (SDI), a comprehensive metric developed by the Institute for Health Metrics and Evaluation (IHME), is designed to gauge the developmental status of countries or regions. It highlights the interrelationship between social progress and population health outcomes. For the GBD 2021 study, after calculating SDI values, they were scaled by a factor of 100 to a range of 0–100. Here, a score of 0 signifies the lowest income, fewest years of schooling, and highest fertility rates, while 100 represents the highest income, most years of schooling, and lowest fertility rates. The 204 countries and territories were classified into five SDI regions: low, low-middle, middle, high-middle, and high in the GBD 2021 (20).

### Statistical analysis

Uncertainty was quantified by sampling from the posterior distributions at each stage of the modeling process and reported as 95% uncertainty intervals (UIs), with the lower and upper bounds corresponding to the 2.5th and 97.5th percentiles, respectively (19). This study employed the Estimated Annual Percentage Change (EAPC) to evaluate trends in prevalence and mortality. EAPCs were derived from a regression model (Y = α + βX + e) applied to ASR over time. A linear regression model was used to compute the 95% confidence interval (CI) for EAPC. The Join-point regression model was utilized to analyze temporal trends in HHD disease burden. This model employs the least-squares approach to quantify rate change patterns. By minimizing the sum of squared residuals between modeled and observed values, it identifies inflection points in trend trajectories. The analysis was executed using Join-point Software, a specialized tool for this methodology. For each sub-interval, we calculated the Annual Percentage Change (APC) to measure the magnitude and direction of trends. The Average Annual Percentage Change (AAPC) over predefined intervals was then computed by weighting segment-specific APCs according to their temporal durations (23, 24). The age-period-cohort (APC) model was employed to examine the distinct impacts of age, period, and cohort on health outcomes, accessible at https://analysistools.cancer.gov/apc/. The age effect estimates the risk of outcomes at different life stages; the period effect captures the influence of temporal trends on outcomes across all age groups; and the cohort effect reflects variations in outcomes among individuals from the same generational groups. The log-linear regression model is specified as: log (Y_i_) = μ + α · age_i_ + β · period_i_ + γ · cohort_i_ + ε, where Y_i_ denotes the HHD prevalence or mortality rate; α, β, and γ represent the coefficients associated with age, period, and cohort effects, respectively; μ is the intercept; and *ε* is the model's residual term. The relative risk (RR) values for each age, period and cohort represent the independent risks in comparison with the reference group [Exp(α–αmean), Exp(β–βmean), Exp(γ–γmean)] (25). We employed decomposition analysis to quantify the contributions of three critical determinants—population aging, demographic transitions, and epidemiological shifts—to the trends in prevalence, mortality, and DALYs between 1990 and 2021. Concurrently, frontier modeling was conducted to assess the relationship between the burden of HHD and sociodemographic development. The gap between a country's observed DALYs rate and the corresponding developmental frontier signifies the unexploited health improvement potential at its current socioeconomic stage. During the ARIMA modeling process, differencing was first applied to stabilize the time-series data. The “auto.arima()” function was then employed to identify the optimal model configuration, leveraging the Akaike Information Criterion (AIC) as the optimization metric (26). We conducted all statistical analyses and visualizations using R software (version 4.4.2).

### Risk factors

GBD 2021 provided a comprehensive assessment of the DALYs and mortality attributable to 87 risk factors, both globally and across regions (27). Among the risk factors linked to HHD are high systolic blood pressure, high Body Mass Index (BMI), alcohol consumption, sodium-rich diets, lead exposure, low temperatures, diet low in fruits, and diet low in vegetables. The detailed definitions of these risk factors, as well as the methodologies used to quantify their contributions to DALYs and mortality, have been extensively described in prior studies (28).

## Result

### Regional change

Globally, there has been a gradual upward trend in the number of HHD prevalence and mortality among WCBA. The number of prevalence has substantially grown from 259,542 in 1990 to 544,544 in 2021, registering a growth of 110% (Table 1). Correspondingly, the mortality number has escalated from 20,198 in 1990 to 25,669 in 2021, marking a 27% increment (Table 2). Although there has been an increase in the number, the rates exhibited markedly distinct trends between 1990 and 2021. Specifically, the prevalence demonstrated an upward trajectory, with an EAPC of 1.31 (1.25,1.37). Conversely, the mortality rate displayed a declining pattern, with an EAPC of −0.51 (−0.59,−0.42). Notably, the regions of East Asia and Western Europe witnessed particularly notable surges. In these areas, the EAPCs reached 2.64 (2.44,2.85) and 2.01 (1.69,2.33), respectively. By contrast, only a limited number of regions, including Eastern Europe and Central Latin America, experienced a reduction in prevalence. The corresponding EAPCs for these regions were −0.91 (−1.11,−0.71) and −0.02 (−0.10,0.05), respectively (Supplementary Table S1). Among the 18 regions across the globe, mortality rates showed a downward trend as time elapsed (Supplementary Table S2). The most substantial decreases were observed in East Asia and the high-income Asia-Pacific, where the EAPCs for mortality were −3.03 (−3.43,−2.63) and −3.75 (−4.36,−3.14), respectively. In Eastern Europe, the quantities of prevalence and mortality, along with their respective rates, manifested a clear downward tendency. Nevertheless, in sub-Saharan Africa and high-income North America, all have been on the rise.

**Table 1:** HHD prevalence among WCBA and trends from 1990 to 2021.

Location	Number (95% UI)			Rate per 100,000 (95% UI)		
	1990	2021	Percentage change (100%)	1990	2021	EAPC (95% CI)
Global	259,542.11 (197,251.36–343,486.71)	544,544.90 (400,850.09–742,840.06)	1.10	19.41 (14.75–25.68)	27.94 (20.57–38.12)	1.31 (1.25,1.37)
High SDI	49,475.25 (37,244.35–65,706.50)	71,910.15 (53,563.63–94,227.94)	0.45	21.82 (16.43–28.98)	29.57 (22.03–38.75)	1.27 (1.18,1.36)
High-middle SDI	36,436.73 (27,684.13–48,007.48)	75,096.03 (55,032.07–105,456.61)	1.06	13.12 (9.97–17.28)	24.61 (18.04–34.56)	2.13 (2.05,2.22)
Middle SDI	89,755.21 (68,765.49–118,689.89)	191,010.70 (140,556.17–262,241.50)	1.13	20.08 (15.38–26.55)	30.88 (22.73–42.40)	1.51 (1.42,1.61)
Low-middle SDI	58,603.28 (44,541.54–77,201.83)	134,297.17 (98,867.69–185,186.99)	1.29	21.47 (16.32–28.29)	26.53 (19.53–36.58)	0.76 (0.74,0.78)
Low SDI	25,033.05 (17,836.36–35,085.42)	71,772.77 (50,590.24–100,631.34)	1.87	22.41 (15.97–31.41)	26.16 (18.44–36.69)	0.61 (0.57,0.66)

**Table 2:** HHD mortality among WCBA and trends from 1990 to 2021.

Location	Number (95% UI)			Rate per 100,000 (95% UI)		
	1990	2021	Percentage change (100%)	1990	2021	EAPC (95% CI)
Global	20,198.91 (12,025.95–25,327.64)	25,669.09 (18,551.15–30,045.34)	0.27	1.51 (0.90–1.89)	1.32 (0.95–1.54)	−0.51 (−0.59, −0.42)
High SDI	1,159.29 (1,081.86–1,237.01)	1,940.50 (1,640.70–2,158.01)	0.67	0.51 (0.48–0.55)	0.80 (0.67–0.89)	1.83 (1.66,2)
High-middle SDI	2,202.00 (1,715.62–2,723.27)	1,490.29 (1,251.87–1,823.87)	−0.32	0.79 (0.62–0.98)	0.49 (0.41–0.60)	−1.84 (−1.98, −1.69)
Middle SDI	7,469.27 (4,524.02–9,048.55)	7,255.26 (5,343.63–8,669.32)	−0.03	1.67 (1.01–2.02)	1.17 (0.86–1.40)	−1.16 (−1.32, −1)
Low-middle SDI	5,518.01 (2,490.17–7,675.71)	8,260.20 (5,617.28–10,103.75)	0.50	2.02 (0.91–2.81)	1.63 (1.11–2.00)	−0.69 (−0.74, −0.65)
Low SDI	3,829.89 (1,487.86–5,410.97)	6,694.59 (3,814.50–8,823.44)	0.75	3.43 (1.33–4.84)	2.44 (1.39–3.22)	−1.32 (−1.44, −1.2)

**Figure 1:**
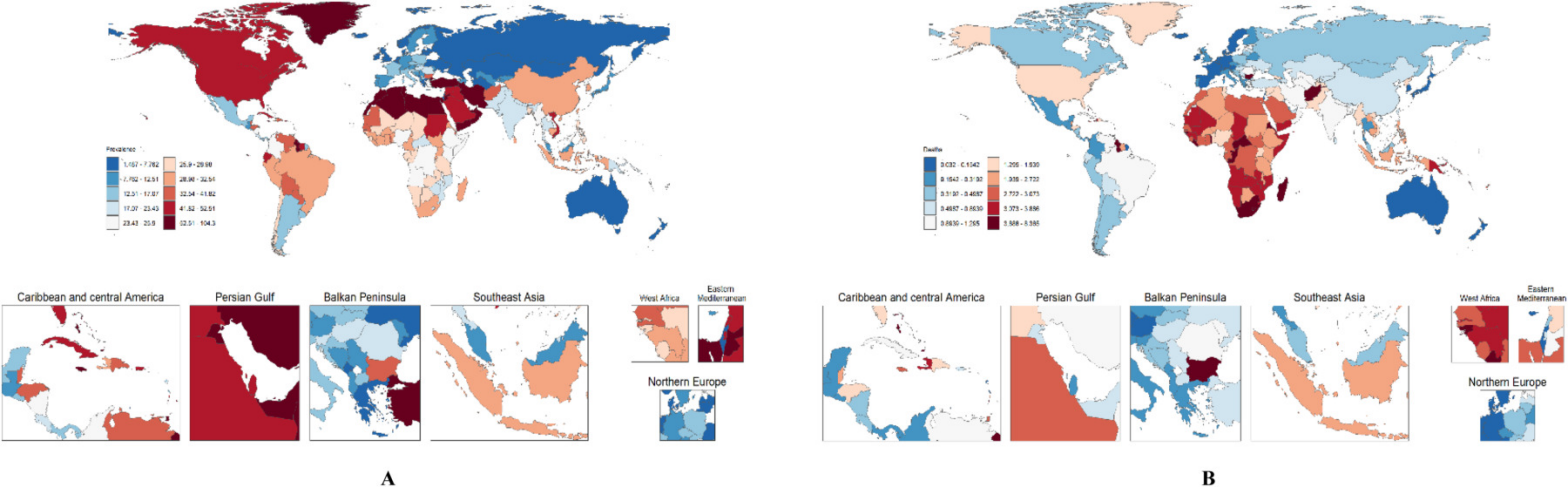
Distribution of HHD in WCBA by 204 countries in 2021. **(A)** prevalence; **(B)** mortality.

Figure 1 indicates the prevalence and mortality distribution of HHD among WCBA by 204 countries in 2021. Supplementary Table S1 illustrates that North Africa and the Middle East, along with high-income North America and the Caribbean, possess relatively elevated prevalence figures. Conversely, Eastern Europe and Australasia exhibit lower prevalence rates. They extend from 2.89(1.83,4.49) cases per 100,000 individuals in Eastern Europe to 58.98(43.72,79.44) cases per 100,000 individuals in North Africa and the Middle East. Southern Sub-Saharan Africa and Oceania have relatively high mortality rates associated with HHD. In contrast, Western Europe and Australasia feature lower mortality. By region, the mortality varies from 0.09/100,000 cases (0.08,0.10) in Australasia to 3.95/100,000 cases (3.12,5.36) in Southern Sub-Saharan Africa (Supplementary Tables S1, S2).

### Age change

Across the global WCBA, the figures for HHD prevalence, mortality, and DALYs exhibited an upward trend as age increased (Supplementary Figure S1). The 15–19 age group registered the lowest values among these metrics. In contrast, the 45–49 age group demonstrated the highest values, which were approximately 9 to 10 times the increment observed in the corresponding values of the 15–19 age group. Notably, there was a decrease in prevalence within the 15–19 age group in the high-middle SDI regions. The age-related percentage change for this decrease was recorded as −0.03 (Supplementary Table S1).

An examination of the global tendency in the prevalence of HHD among WCBA from 1990 to 2021 reveals that the prevalence has been on the rise across all age brackets. Specifically, within high SDI regions, the values of the EAPC were especially notable in the 25–29 and 30–34 age groups. These values reached 1.91 (1.62,2.2) and 1.95 (1.65,2.25), respectively. In addition, the prevalence within the 25–49 age group also demonstrated an upward trajectory. Throughout the past 32 years, the global rates of prevalence, mortality, and DALYs have escalated with advancing age. Among these, the most substantial increase was observed in the 45–49 age group (Supplementary Figure S2).

HHD prevalence rises steadily from the 15–19 age group, but it trends upward distinctly in the 45–49 age group (Supplementary Figure S3A). The relative risk (RR) fell from 1.017 (0.783,1.320) in 1994 to 0.904 (0.727,1.124) in 2019 (Supplementary Figure S3B). Among birth cohorts, prevalence was lower in earlier years. Cohort risk was high in the 1947–1952 birth cohort (RR = 1.487; 1.094,2.021), then fluctuated after declining (Supplementary Figure S3C). Age-specific mortality rates increased slowly. For those under 45, RR values first rose gradually, then faster [R Age (45–49) = 0.001; 0.001,0.002] (Supplementary Figure S3D). Mortality rates per period have declined yearly since 1994 [RR period (1994) = 1.251; 0.406,3.850], hitting the lowest in 2019 [RR period (2019) = 0.741; 0.265,2.071] (Supplementary Figure S3E). Early birth cohorts had a stronger impact on HHD mortality. RR values were 1.844–2.442 until 1947–1952, then dropped from 1.552 (1952–1957 cohort) to 0.720 (1997–2002 cohort) (Supplementary Figure S3F). DALYs rates followed the mortality trend but with more significant changes. For age cohorts, there was a continuous increase from 15 to 19 [R Age (15–19) = 0.003; 0.002, 0.004] to 45–49 [R Age (45–49) = 0.044; 0.040,0.049] (Supplementary Figure S3G). Both period and early birth cohorts trended downward. The RR for the period cohort decreased from 1.240 in 1994 to 0.746 in 2019 (Supplementary Figure S3H), and for the early birth cohort, from 1.552 (1952–1957) to 0.720 (1997–2002) (Supplementary Figure S3I).

### Time change

Supplementary Figure S4 vividly illustrates the temporal dynamic trends in the global rate and number of prevalence, mortality, and DALYs of HHD among WCBA from 1990 to 2021. These crucial indicators exhibit distinct fluctuating patterns throughout the timeline. In particular, the disease prevalence has been on a gradual upward trajectory. Conversely, both the mortality rate and the DALYs rate have predominantly demonstrated a downward tendency.

The joint point regression analysis reveals that, commencing from 1990, the prevalence has been experiencing an annual increase. Notably, a particularly substantial upsurge was witnessed during the period from 2001 to 2004, where the annual percentage change (APC) reached a value of 2.86. Conversely, between 2019 and 2021, a downward trend in the prevalence of HHD was detected, with an APC recorded at −0.28 (Supplementary Figure S5A). Shifting the focus to the fluctuations in mortality rates, from 1990 to 2006, there was a notable decrease. Subsequently, between 2003 and 2006, the mortality rate stabilized, with an APC of −1.83 for that specific interval (Supplementary Figure S5B). In comparison to the changes in prevalence and mortality, the alterations in the rates of DALYs were more striking. From 1990 to 2011, the DALYs rate dropped sharply, and the most significant decline occurred between 2003 and 2006, during which the APC was −1.62. Intriguingly, though, between 2011 and 2017, the DALYs rate deviated from the previous downward trajectory (Supplementary Figure S5C).

Supplementary Table S2 provides a detailed account of the average annual percentage changes (AAPCs) in HHD over the past three decades, specifically concerning prevalence, mortality, and DALYs rates. The information presented in the table reveals that between 1990 and 2021, the global prevalence witnessed an increment of 1.20 (1.16,1.23). In contrast, the mortality rate experienced a decrease of −0.43 (−0.54,−0.32), and the DALYs rate saw a decline of −0.44 (−0.54,−0.33).

### Relationship between HHD burden and SDI

In 2021, the middle SDI regions exhibited the highest prevalence, registering 30.88(22.73,42.40) cases per 100,000 individuals. Conversely, the low SDI regions were at the forefront in terms of mortality and DALYs, with 2.44(1.39,3.22) cases per 100,000 individuals and 125.39(73.95,165.06) DALYs per 100,000 individuals. Moreover, the rates of these relevant indicators in these regions were roughly 1.1 to 1.8 times the global average rate (Supplementary Figure S6). Throughout the 21 regions globally, the burden of HHD remained relatively constant when the SDI value was below 0.5. Conversely, it increased incrementally when the SDI value surpassed 0.5. Regarding the geographical distribution, the burden in areas like South Asia and Southern Sub-Saharan Africa was higher than anticipated. In contrast, the burden in regions such as Eastern Europe, Australasia, and the high-income Asia-Pacific area was lower than what was expected. HHD-related death and DALY burdens were similar and consistent (Supplementary Figure S7).

Over the past three decades, age structure and epidemiological changes were responsible for 21.26% and 28.39% of the factors that contributed to the augmented prevalence burden, and population growth had the greatest influence, accounting for 50.38%. Notably, in the low-middle SDI regions, the contribution of population growth was the most substantial (96.62%), Additionally, the age structure within the middle SDI regions exerted a most pronounced negative impact (−17.94%) (Supplementary Figure S8A). Regarding the mortality burden (Supplementary Figure S8B), population growth and age structure contributed 294.11% and 172.83%, respectively. Among these, the most significant contribution from population growth (226.38%) was observed in the low-middle SDI regions. Conversely, the effect of epidemiological changes was negative (−366.94%), and most evident in the low-middle SDI regions (−186.08%). At the same time, the DALYs burden is similar to mortality (Supplementary Figure S8C). Population growth and age structure contributed 233.79% and 33.51%. The most significant contribution from population growth (159.61%) was found in the low SDI regions. Moreover, the impact of epidemiological changes was negative (−167.37%), and most prominent in the low-middle SDI regions (−130.7%).

The result of the Frontier analysis reveals that among the 15 countries exhibiting the largest effective variance, France, Australia, and Greenland are notable examples. In contrast, countries and regions characterized by a low SDI value (<0.5) and a low effective variance include Yemen, Papua New Guinea, and Timor-Leste. Similarly, countries with a high SDI (>0.85) and relatively advanced levels of development that also possess a large effective variance include Canada, Germany, and Iceland, among others. This indicates that countries or regions with higher SDI values have a greater capacity for making progress in reducing the burden of HHD (Supplementary Figure S9).

### HHD-related risk factors and projected HHD prevalence and mortality by 2040

**Figure 2:**
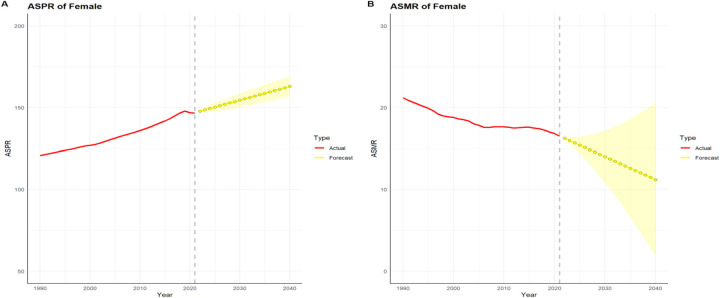
HHD projection of age-standardized rates of prevalence (ASPR) and mortality (ASMR) from 2022 to 2040. **(A)** ASPR; **(B)** ASMR.

An ARIMA model was employed to forecast the age-standardized rates of HHD over the subsequent 19-year period, extending up to 2040. Based on the projections generated by this model, the prevalence is anticipated to rise from 147.77 cases per 100,000 individuals in 2022 to 162.86 cases per 100,000 individuals by 2040. Conversely, the mortality is predicted to decline from 16.24 deaths per 100,000 individuals in 2022 to 11.2 deaths per 100,000 individuals in 2040 (Figure 2). Collectively, the model-based forecasts imply that, over the long term, the trend of change from 2022 to 2040 is roughly similar to that from 1990 to 2021.

**Figure 3:**
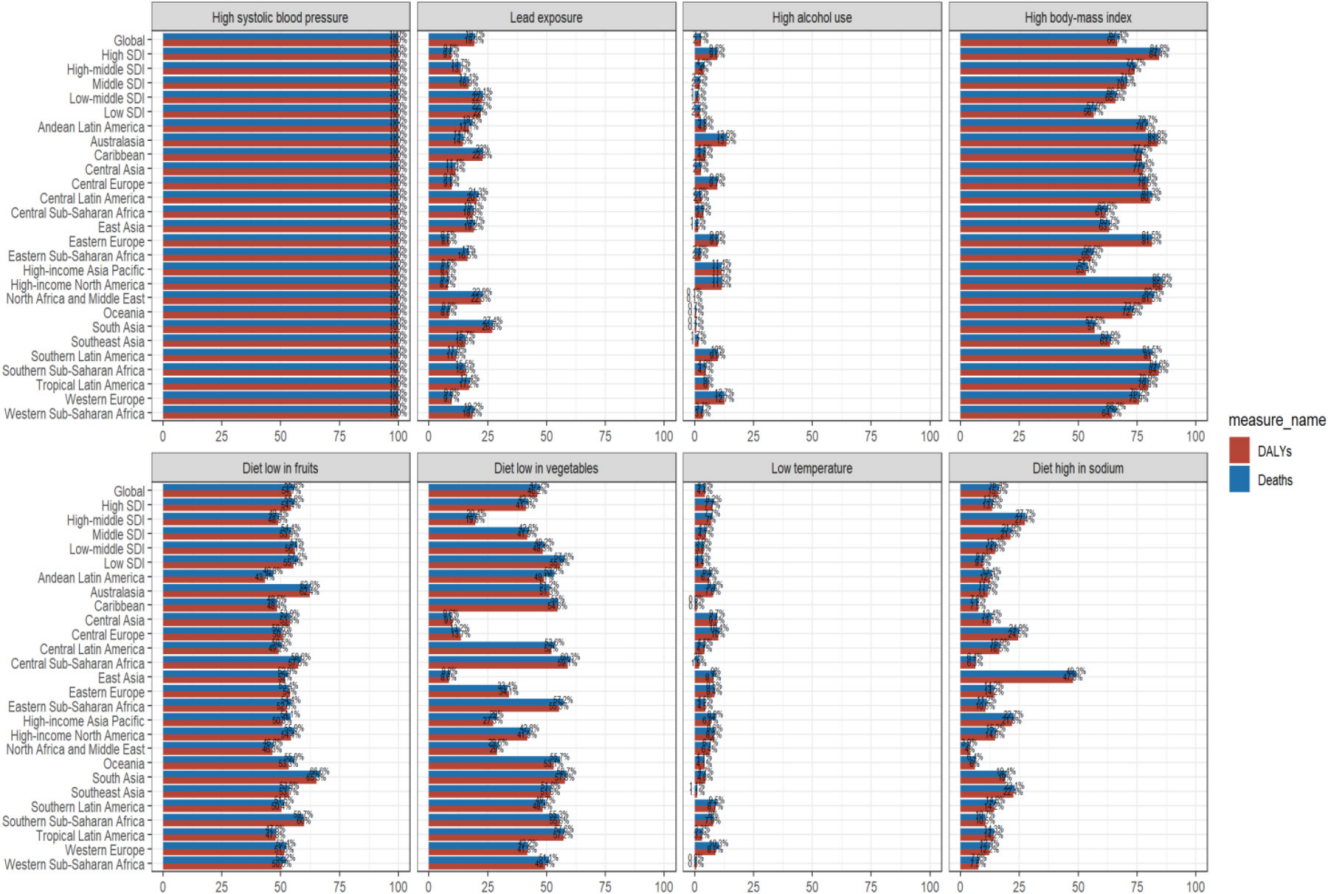
Proportion (%) in WCBA of HHD deaths and DALYs due to risk factors in 2021.

Since HHD is a progressive stage of hypertension, the impact is 100% on mortality and DALYs. Lead exposure, high alcohol use, and low temperature have smaller effects on mortality and DALYs. When examined from the perspective of mortality, as much as 55.8% can be attributed to variations in diet low in fruits. Additionally, 47.2% are linked to diet low in vegetables, and 67.4% are associated with a high BMI. In terms of DALYs, 54.7% can be ascribed to disparities in diet low in fruits, 46.2% are due to diet low in vegetables, and another 66.7% are related to a high BMI. Significantly, high BMI emerged as the most crucial risk factors for triggering HHD. These factors impact mortality and DALYs in about half the cases. Notably, High-sodium diets in East Asia far exceed those in other regions (Figure 3).

## Discussion

Manandhar and his colleagues formulated the notion of “ensuring health equity for every individual, protecting health and promoting overall well-being, all the while strengthening gender equality and empowering women”. This concept delineates the objective of decreasing the mortality rate among the global population and alleviating the disease burden by the year 2030 (29). An in-depth and comprehensive understanding of the prevalence is essential to assess the likelihood of achieving health goals. However, there is a lack of comprehensive literature analysis of prevalence, mortality, and DALYs in WCBA in different countries and regions around the globe, and previous studies have focused on specific age groups or countries (30–32). Therefore, we believe there is a need to enhance and update timely data on the burden of HHD in the global population of WCBA to enable policymakers to be informed and develop effective prevention and control strategies. This study provides a comprehensive estimate of prevalence, mortality, and DALYs over the past 32 years at the global level.

The results of the study indicate that in 2021, there will be 259,000 prevalence and 20,000 deaths among WCBA. The increasing prevalence of hypertension, one of the important risk factors, is further contributing to the rising prevalence (33). HHD is not merely a significant issue in the control and treatment of hypertension; it also poses a crucial and formidable public health challenge (6, 34). We discovered that, from 1990 to 2021, there was an overall upward prevalence trend in the WCBA. This may be since HHD detection methods have been upgraded, allowing more cases to be detected. In contrast, mortality and DALYs values have decreased. This strongly suggests that the prevention and treatment over the past three decades have had a positive impact on a global scale.

A rising trend in the cases of prevalence over the past 32 years globally with age, with the highest increase observed in the 45–49 age group, particularly in middle SDI regions. This phenomenon may be the result of a combination of biological, genetic, psychological, and social factors. Women aged 45–49 experience hormonal fluctuations during perimenopause and menopause, especially fluctuations in estrogen and progesterone, which may play a key role in the development of HHD in WCBA. A study of postmenopausal women taking estrogen/progestin therapy found that after long-term treatment, women had fewer cardiovascular events than placebo controls (35). Relevant studies have found that the prevalence of hypertension increases with age, and below 50 years old, it is higher in men than in women. Conversely, for those above 50 years of age, the condition is more prevalent in females (36). The reason underlying this disparity in the prevalence of hypertension between genders is still not fully understood. However, it is widely recognized that males tend to develop cardiovascular diseases at an earlier age compared to females. Menstruation-related problems specific to women may have a potential impact on the cardiovascular system.

We discerned striking disparities and evolving trends in the burden of HHD that were not only pronounced but also reflected the unique health characteristics and socio-economic factors influencing each geographical area, highlighting the complexity of managing and addressing HHD on a global scale. Among the 21 GBD regions, the number and rate of prevalence, as well as those of deaths, have declined significantly in Eastern Europe, but have increased in sub-Saharan Africa and high-income North America. Notably, elevated prevalence rates of hypertension have been detected in specific countries situated within Central Asia, Southern Africa, and the Caribbean regions. This observation appears to be at odds with the findings of our studies (37–39). Interestingly, the mortality rate in the high-income North America is higher than that in other regions (EAPC +2.9). High-salt, high-sugar, and high-fat diets exacerbate hypertensive disorders while failing to provide timely control management of hypertension are key to the problem (40, 41). Although HHD represents a form of target organ damage resulting from hypertension, its prevalence does not invariably align with that of hypertension. The relationship between the two is more nuanced and complex. Intriguingly, the findings from two separate contemporaneous studies lend credence to our conclusion (42, 43). The study notes that middle SDI regions had the highest prevalence (30.88/100,000), while low SDI regions had the highest mortality (2.44/100,000), a phenomenon that can be attributed to the following several main reasons: (1) Medical Infrastructure: In areas with good medical facilities, high blood pressure is managed better, and HHD treatment is more effective. But in places with weak medical set-ups, high blood pressure management has problems and treatment doesn't work well (44). This is also the reason why, in low SDI regions, although the prevalence rate is relatively low, the mortality rate is the highest; (2) Economy: How much money people have impacted their health habits and access to healthcare. Poor people often can't afford healthy food or proper healthcare, so they get sick more easily (45); (3) Public Health and Awareness: Regions with good health policies and people who know more about health can prevent and control diseases better (42). Health care policies in middle SDI regions may be far less favorable than in high SDI regions, which leads to higher prevalence rates. In summary, differences in healthcare infrastructure, economic factors, and differences in public health policies and health awareness combine to contribute to differences in the burden of HHD in different regions of the world.

The 2040 projection indicates a paradoxical trend: while the prevalence of cardiovascular diseases is expected to rise, mortality rates are projected to decline. This trend suggests a critical demographic shift in health outcomes—from fatal events to increased disability, with conditions like heart failure emerging as disease burden (6). It is crucial to concentrate on implementing a series of targeted initiatives to effectively address the relevant risk factors. First, efforts should be made to control BMI levels, as maintaining an appropriate BMI is essential for overall health (1, 46). Additionally, curbing high sodium intake from diets is of great significance, given that excessive sodium consumption can pose risks to cardiovascular health (47). Equally important is the reduction of alcohol consumption, as excessive drinking is known to contribute to various health problems (37, 48). By focusing on these aspects, we can more comprehensively manage and mitigate potential health risks. In some middle SDI and East Asian regions, the number of obese individuals is rapidly increasing, highlighting the need for vigilance concerning the risks posed by a high body mass index to HHD (49). Moreover, the result of this study shows that the effect of high sodium diet on HHD is more pronounced in certain regions, such as East Asia. Therefore, moderately cutting down on salt intake and maintaining a nutritious diet can effectively lower the mortality rates caused by cardiovascular complications (50). Alcohol has the potential to trigger cardiac fibrillar hyperplasia, a pathological process that disrupts the normal structure and function of the heart muscle; therefore, the implementation of specific alcohol intervention initiatives is expected to be effective in reducing the burden of disease due to hypertension or HHD, helping to improve public health and reducing pressure on the healthcare system (51). Given the relative lack of health care resources in low-income and middle-income countries, we earnestly hope that the governments and health care organizations of these countries will take active action (37, 47). Through a series of effective measures, such as improving health conditions to build a solid foundation for health, vigorously strengthening early screening for early detection of the disease, and widely promoting the adoption of a healthy lifestyle to enhance people's health literacy, the heavy burden brought about by HHD can be effectively alleviated.

This study has multiple limitations. To begin with, the data sourced from GBD is predominantly dependent on modelled data. This is because collaborators employ a variety of statistical modelling techniques, particularly in nations where primary data is scarce. Secondly, precisely defining the burden of HHD from a single perspective is intricate. As a consequence, the definitions of disability weights remain rather incomplete. Moreover, it is crucial to recognize the time-lagged characteristic of GBD data. Consequently, on one hand, there is a requirement for further refinement of scales and coefficients related to patients' disability. On the other hand, supplementary real-world research is essential to verify the results, thereby enabling a more precise and comprehensive evaluation.

## Conclusion

Overall, during the past 32 years, the global burden of HHD among WCBA has experienced an upward trajectory. About socioeconomic aspects, irrespective of geographical regions or individual nations, the burden shows a positive correlation with SDI. Notably, the most pronounced and substantial growth in the disease burden has been witnessed in low SDI regions. This escalating trend can potentially be ascribed to the uneven availability of preventive, diagnostic, and treatment resources across diverse global regions. Regarding age-related patterns, over the past 32-year span, the 45–49 age group has seen the most significant increase. Concurrently, there is an urgent need to enhance resource allocation in the healthcare domain, particularly during adulthood. This is crucial to mitigate the risk for successive younger birth cohorts and all age groups as time progresses.

## Data Availability

Publicly available datasets were analyzed in this study. This data can be found here: https://vizhub.healthdata.org/gbd-results.
