# Supplementary material for: Burden of hypertensive heart disease among women of childbearing age in global, regional, and national regions from 1990 to 2021 and projection until 2040
Source: Front Glob Womens Health. 2025 Jul 11;6:1600340. doi: 10.3389/fgwh.2025.1600340 (PMC12289639; doi:10.3389/fgwh.2025.1600340)
Supplement: Supplementary file 1 [file Datasheet1.docx]

| **Location** | **Number (95% UI)** | | | **Rate per 100,000 (95% UI)** | | | |
| --- | --- | --- | --- | --- | --- | --- | --- |
|  | **1990** | **2021** | **Percentage change (100%)** | | **1990** | **2021** | **EAPC (95% CI)** |
| **Central Europe** | 3,554.33 (2,744.02-4,565.51) | 4,247.90 (3,132.56-5,622.20) | 0.20 | | 11.57 (8.93-14.87) | 16.49 (12.16-21.83) | 1.27 (1.18,1.36) |
| **Eastern Europe** | 2,067.15 (1,467.80-2,951.04) | 1,394.73 (882.37-2,164.67) | -0.33 | | 3.74 (2.65-5.34) | 2.89 (1.83-4.49) | -0.91 (-1.11, -0.71) |
| **Western Europe** | 6,763.73 (4,970.19-9,041.75) | 9,695.46 (6,973.08-13,434.30) | 0.43 | | 7.08 (5.20-9.46) | 10.41 (7.48-14.42) | 2.01 (1.69,2.33) |
| **Caribbean** | 2,676.10 (2,032.58-3,450.69) | 4,872.77 (3,562.76-6,701.70) | 0.82 | | 28.71 (21.81-37.02) | 40.51 (29.62-55.71) | 1.22 (1.14,1.3) |
| **Central Sub-Saharan Africa** | 2,563.03 (1,628.31-3,962.91) | 7,970.57 (5,090.83-12,401.71) | 2.11 | | 20.73 (13.17-32.06) | 24.41 (15.59-37.98) | 0.53 (0.51,0.56) |
| **Eastern Sub-Saharan Africa** | 10,293.61 (6,967.63-14,970.28) | 29,031.41 (19,409.53-42,488.07) | 1.82 | | 23.86 (16.15-34.69) | 27.11 (18.12-39.67) | 0.48 (0.43,0.52) |
| **Southern Sub-Saharan Africa** | 2,603.15 (1,739.77-3,868.10) | 5,969.76 (3,884.84-8,953.28) | 1.29 | | 19.58 (13.09-29.10) | 27.50 (17.89-41.24) | 1.02 (0.95,1.09) |
| **Western Sub-Saharan Africa** | 10,971.55 (7,541.03-15,942.19) | 32,917.07 (22,032.74-48,555.98) | 2.00 | | 25.16 (17.29-36.56) | 27.46 (18.38-40.50) | 0.52 (0.38,0.67) |
| **North Africa and Middle East** | 37,561.69 (29,149.11-48,090.48) | 93,982.99 (69,660.22-126,576.63) | 1.50 | | 48.08 (37.31-61.56) | 58.98 (43.72-79.44) | 0.71 (0.62,0.81) |
| **South Asia** | 42,286.65 (31,513.46-57,706.40) | 105,079.26 (74,760.06-151,428.31) | 1.48 | | 16.59 (12.36-22.64) | 21.27 (15.13-30.64) | 0.9 (0.85,0.95) |
| **East Asia** | 46,680.42 (34,578.35-63,648.36) | 98,553.76 (70,156.21-140,847.70) | 1.11 | | 14.00 (10.37-19.09) | 29.78 (21.20-42.56) | 2.64 (2.44,2.85) |
| **Central Asia** | 1,707.27 (1,233.85-2,269.81) | 2,782.39 (1,991.29-3,803.63) | 0.63 | | 10.18 (7.35-13.53) | 11.47 (8.21-15.68) | 0.61 (0.51,0.72) |
| **Southeast Asia** | 25,714.43 (19,953.86-33,403.25) | 53,440.94 (39,969.17-72,220.15) | 1.08 | | 21.40 (16.60-27.79) | 29.17 (21.82-39.42) | 1.03 (0.98,1.07) |
| **High-income Asia Pacific** | 8,654.74 (6,274.08-11,803.60) | 7,380.01 (5,193.86-10,344.10) | -0.15 | | 18.92 (13.72-25.81) | 19.40 (13.65-27.19) | 0 (-0.14,0.14) |
| **High-income North America** | 30,316.23 (22,626.33-40,894.44) | 42,901.87 (31,026.22-56,925.24) | 0.42 | | 40.77 (30.43-54.99) | 51.06 (36.93-67.75) | 1.09 (0.98,1.21) |
| **Andean Latin America** | 3,037.85 (2,274.56-3,919.17) | 6,233.83 (4,510.31-8,447.52) | 1.05 | | 32.03 (23.98-41.32) | 35.72 (25.84-48.40) | 0.58 (0.48,0.68) |
| **Southern Latin America** | 1,842.40 (1,316.77-2,445.82) | 3,155.96 (2,208.48-4,494.29) | 0.71 | | 14.87 (10.63-19.74) | 18.11 (12.67-25.79) | 0.88 (0.79,0.96) |
| **Central Latin America** | 8,398.40 (6,426.06-10,981.93) | 14,074.76 (10,001.39-19,731.62) | 0.68 | | 20.04 (15.33-26.20) | 20.64 (14.67-28.93) | -0.02 (-0.1,0.05) |
| **Tropical Latin America** | 11,305.75 (8,512.78-14,975.89) | 19,686.53 (13,811.56-28,260.96) | 0.74 | | 28.34 (21.34-37.54) | 32.48 (22.79-46.63) | 0.46 (0.44,0.48) |
| **Australasia** | 240.54 (170.14-331.61) | 413.13 (282.60-597.87) | 0.72 | | 4.48 (3.17-6.18) | 5.72 (3.91-8.28) | 1.04 (0.91,1.18) |
| **Oceania** | 303.09 (224.56-398.30) | 759.82 (545.74-1,061.90) | 1.51 | | 19.51 (14.45-25.63) | 21.89 (15.72-30.59) | 0.35 (0.29,0.41) |

**Table S1 HHD prevalence among WCBA and trends from 1990 to 2021**

| **Location** | **Number (95% UI)** | | | **Rate per 100,000 (95% UI)** | | | |
| --- | --- | --- | --- | --- | --- | --- | --- |
|  | **1990** | **2021** | **Percentage change (100%)** | | **1990** | **2021** | **EAPC (95% CI)** |
| **Central Europe** | 283.88 (270.06-305.73) | 205.67 (176.66-234.73) | -0.28 | | 0.92 (0.88-1.00) | 0.80 (0.69-0.91) | -0.21 (-0.37, -0.04) |
| **Eastern Europe** | 225.33 (213.76-243.35) | 202.59 (170.62-237.78) | -0.10 | | 0.41 (0.39-0.44) | 0.42 (0.35-0.49) | -1.01 (-2.1,0.09) |
| **Western Europe** | 179.85 (171.69-188.45) | 120.21 (115.28-124.29) | -0.33 | | 0.19 (0.18-0.20) | 0.13 (0.12-0.13) | -0.58 (-0.78, -0.38) |
| **Caribbean** | 175.63 (122.65-236.13) | 285.65 (191.24-411.04) | 0.63 | | 1.88 (1.32-2.53) | 2.37 (1.59-3.42) | 1.02 (0.92,1.12) |
| **Central Sub-Saharan Africa** | 469.89 (170.16-758.31) | 1,058.50 (494.52-1,682.70) | 1.25 | | 3.80 (1.38-6.13) | 3.24 (1.51-5.15) | -0.56 (-0.64, -0.48) |
| **Eastern Sub-Saharan Africa** | 2,087.25 (710.21-2,868.31) | 3,024.33 (1,593.15-3,989.88) | 0.45 | | 4.84 (1.65-6.65) | 2.82 (1.49-3.73) | -2.09 (-2.28, -1.91) |
| **Southern Sub-Saharan Africa** | 582.79 (450.75-691.39) | 858.53 (676.53-1,164.84) | 0.47 | | 4.38 (3.39-5.20) | 3.95 (3.12-5.36) | 0.31 (-0.32,0.96) |
| **Western Sub-Saharan Africa** | 1,464.69 (821.02-2,038.67) | 2,834.40 (1,401.41-3,839.63) | 0.94 | | 3.36 (1.88-4.68) | 2.36 (1.17-3.20) | -1.26 (-1.42, -1.11) |
| **North Africa and Middle East** | 2,213.06 (1,025.78-3,093.93) | 3,252.03 (2,153.33-4,222.98) | 0.47 | | 2.83 (1.31-3.96) | 2.04 (1.35-2.65) | -1.03 (-1.21, -0.84) |
| **South Asia** | 3,330.35 (1,049.17-5,004.26) | 5,615.68 (3,552.02-7,557.47) | 0.69 | | 1.31 (0.41-1.96) | 1.14 (0.72-1.53) | -0.45 (-0.54, -0.36) |
| **East Asia** | 4,596.42 (2,582.85-5,980.62) | 1,841.76 (1,117.21-2,953.10) | -0.60 | | 1.38 (0.77-1.79) | 0.56 (0.34-0.89) | -3.03 (-3.43, -2.63) |
| **Central Asia** | 151.42 (132.37-171.42) | 217.90 (176.20-278.39) | 0.44 | | 0.90 (0.79-1.02) | 0.90 (0.73-1.15) | -0.74 (-1.47, -0.02) |
| **Southeast Asia** | 2,455.65 (1,177.10-3,303.47) | 3,510.48 (2,174.74-4,422.69) | 0.43 | | 2.04 (0.98-2.75) | 1.92 (1.19-2.41) | -0.11 (-0.17, -0.05) |
| **High-income Asia Pacific** | 188.28 (143.22-210.42) | 45.78 (41.10-62.12) | -0.76 | | 0.41 (0.31-0.46) | 0.12 (0.11-0.16) | -3.75 (-4.36, -3.14) |
| **High-income North America** | 562.76 (550.04-578.91) | 1,410.79 (1,249.05-1,481.58) | 1.51 | | 0.76 (0.74-0.78) | 1.68 (1.49-1.76) | 2.9 (2.51,3.3) |
| **Andean Latin America** | 100.13 (81.23-120.01) | 91.12 (68.66-125.66) | -0.09 | | 1.06 (0.86-1.27) | 0.52 (0.39-0.72) | -1.99 (-2.37, -1.6) |
| **Southern Latin America** | 81.38 (76.14-86.76) | 67.16 (62.45-71.86) | -0.17 | | 0.66 (0.61-0.70) | 0.39 (0.36-0.41) | -1.51 (-1.76, -1.26) |
| **Central Latin America** | 291.19 (278.10-305.91) | 296.41 (229.46-358.65) | 0.02 | | 0.69 (0.66-0.73) | 0.43 (0.34-0.53) | -1.81 (-2.22, -1.39) |
| **Tropical Latin America** | 697.79 (672.49-724.81) | 615.41 (541.89-651.45) | -0.12 | | 1.75 (1.69-1.82) | 1.02 (0.89-1.07) | -1.03 (-1.21, -0.84) |
| **Australasia** | 6.22 (5.79-6.72) | 6.29 (5.72-6.86) | 0.01 | | 0.12 (0.11-0.13) | 0.09 (0.08-0.10) | -1 (-1.25, -0.76) |
| **Oceania** | 54.96 (23.76-95.68) | 108.39 (55.24-169.95) | 0.97 | | 3.54 (1.53-6.16) | 3.12 (1.59-4.90) | -0.52 (-0.61, -0.43) |

**Table S2 HHD mortality among WCBA and trends from 1990 to 2021**

| **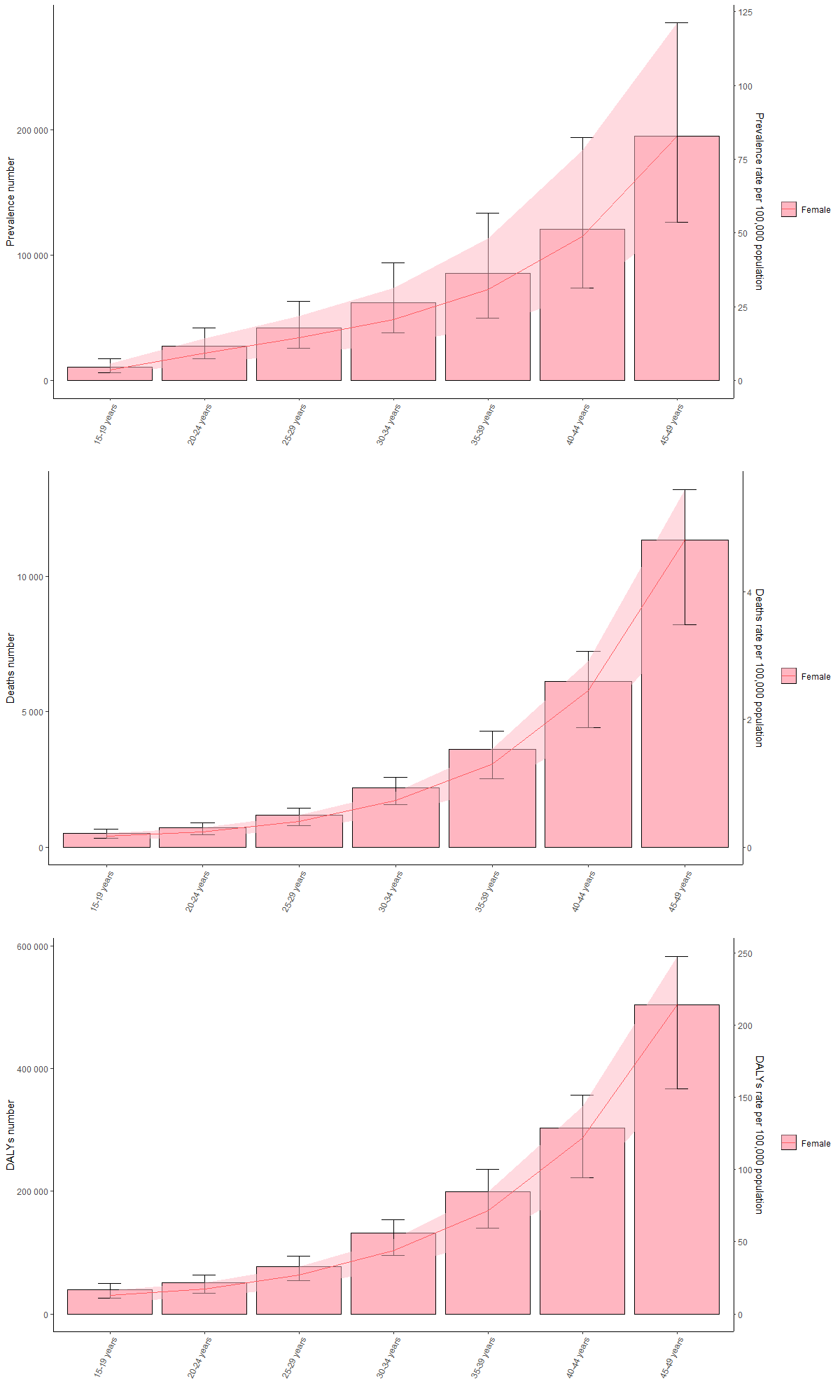**  **Figure S1 Global burden of HHD by age group in 2021; bar graphs are totals; line graphs are rates.** |
| --- |
|  |
|  |

| **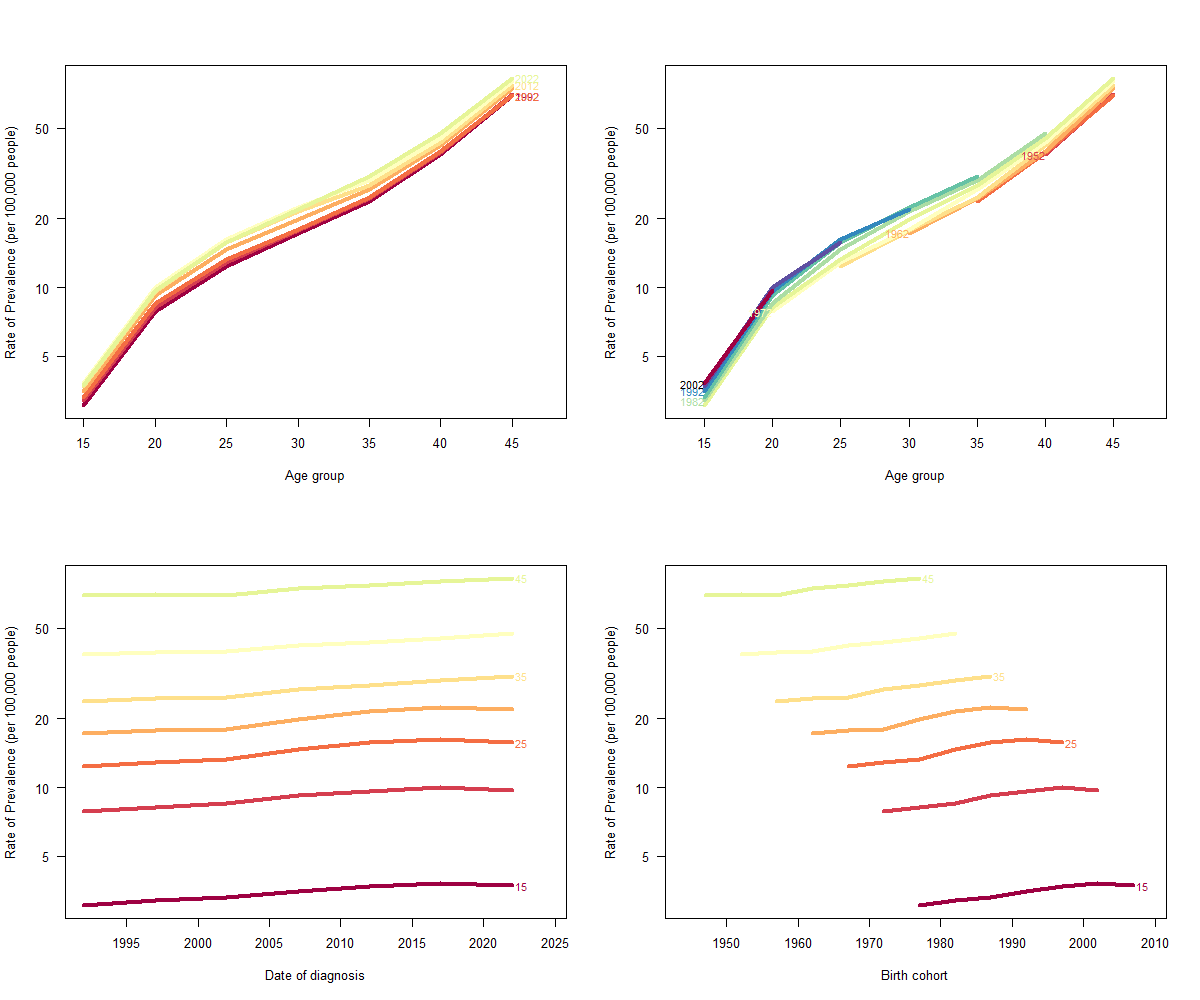A** | | |  |
| --- | --- | --- | --- |
| **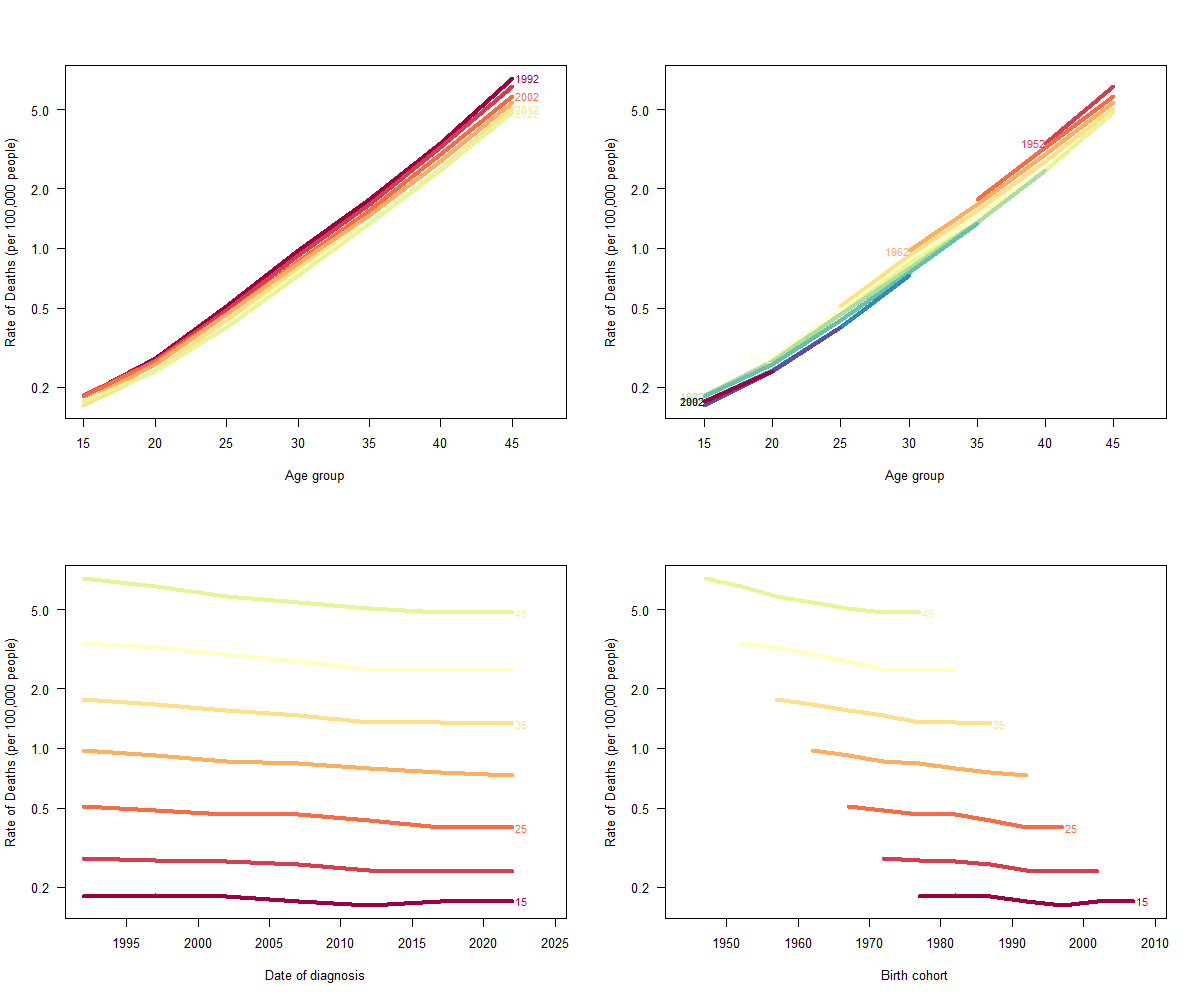B** | | |  |
| **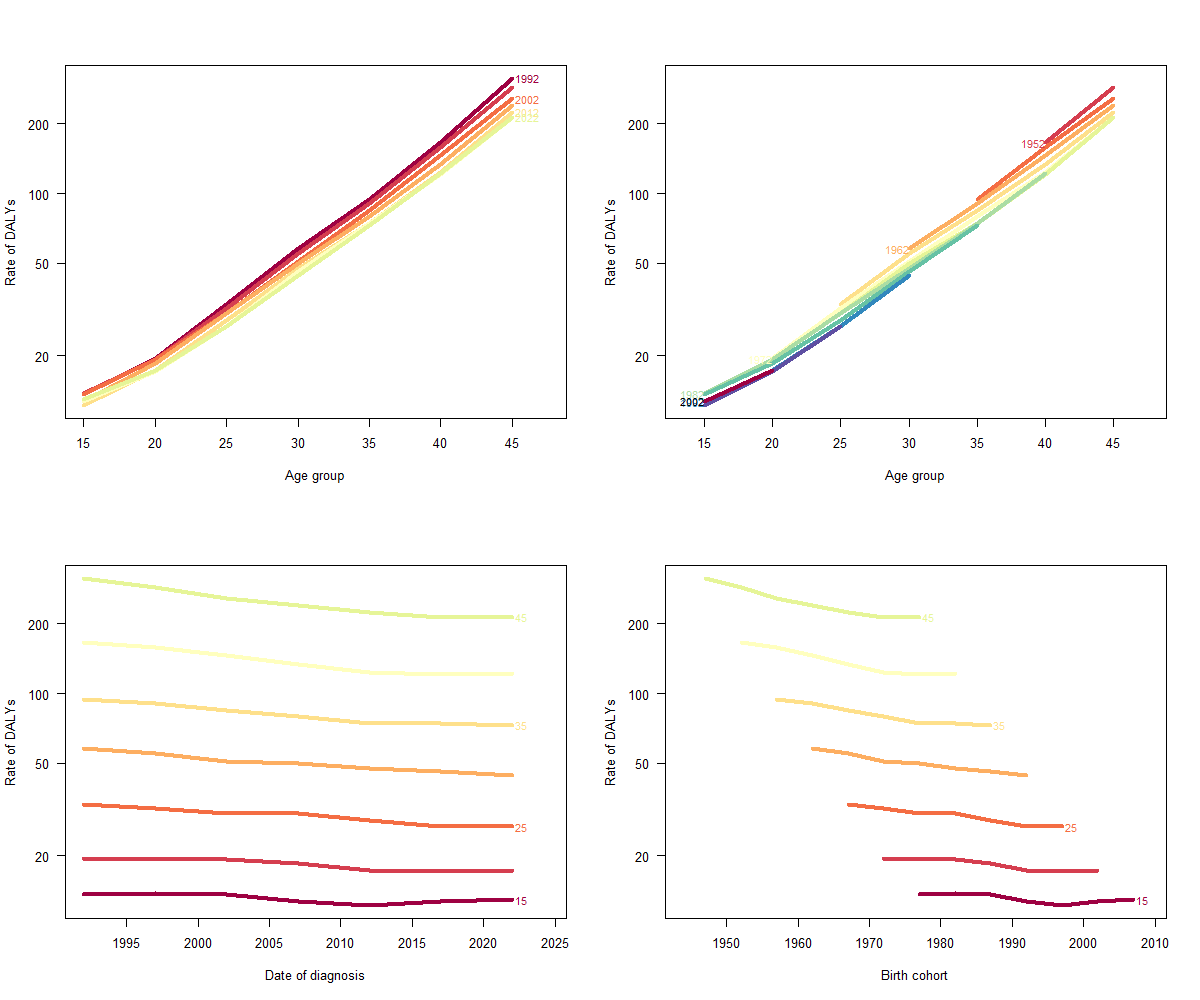C** | | |  |
| **Figure S2 Age, period, and cohort trends in global HHD prevalence, mortality, and DALYs. (A) prevalence; (B) mortality; (C) DALYs.** | | |  |
| **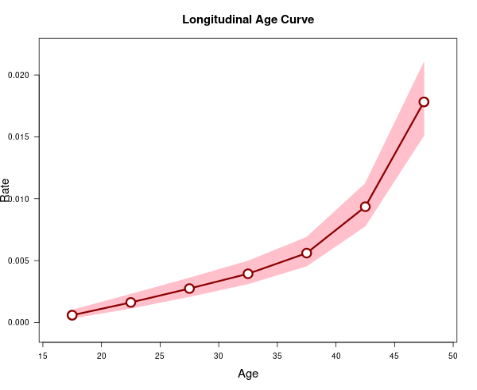A** | **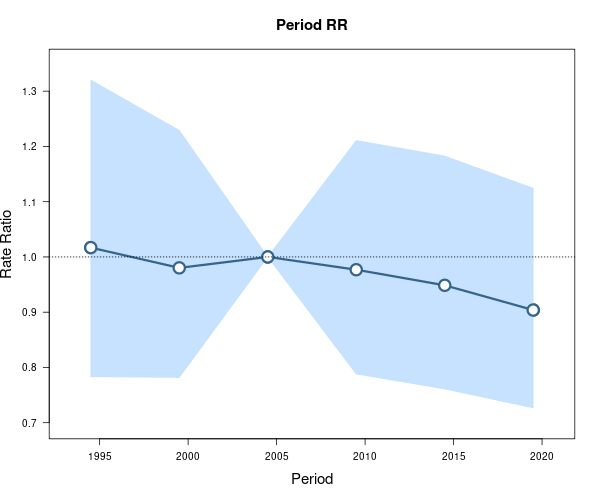B** | **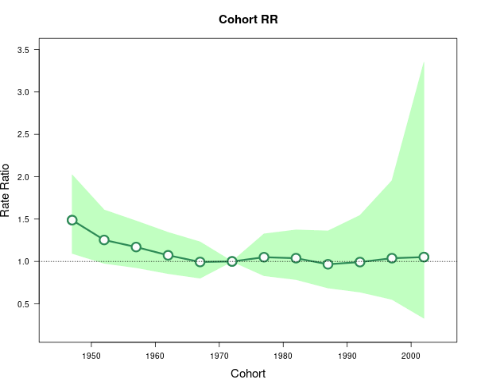C** | |
| **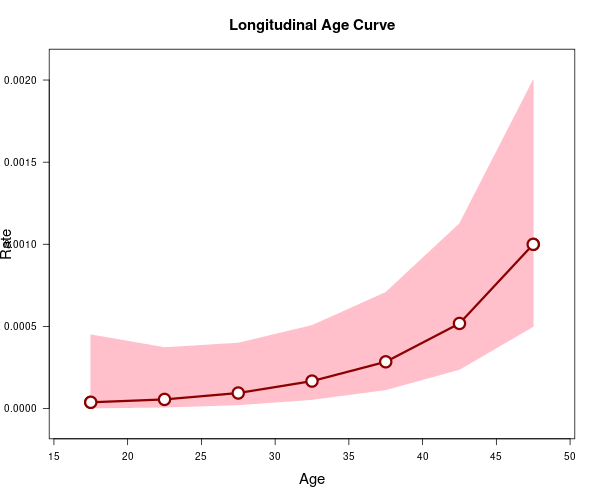D** | **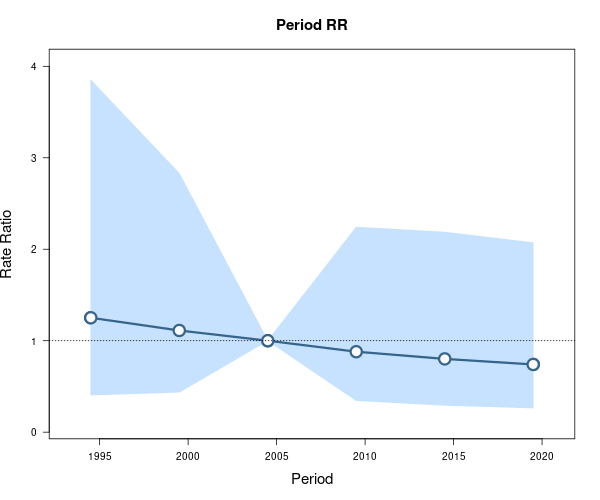E** | **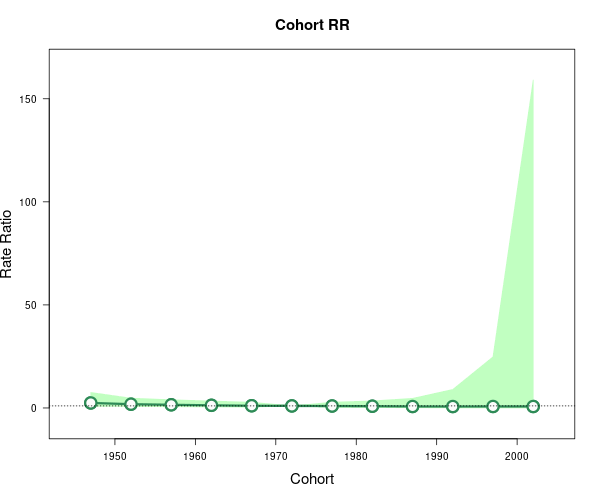F** | |
| **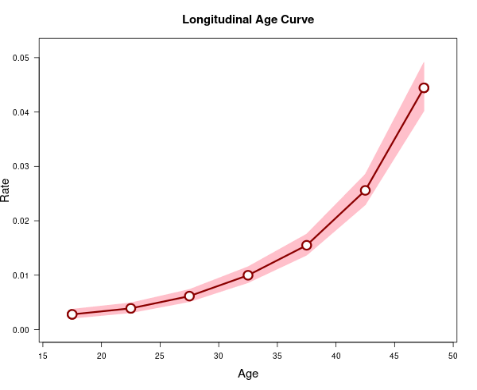G** | **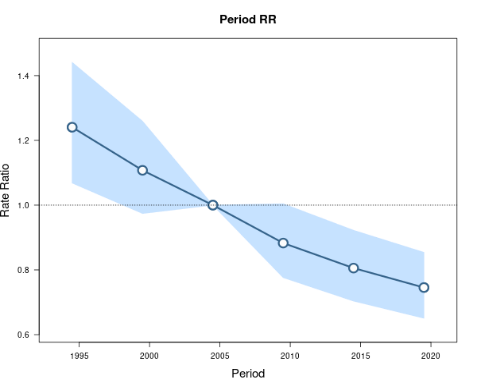H** | **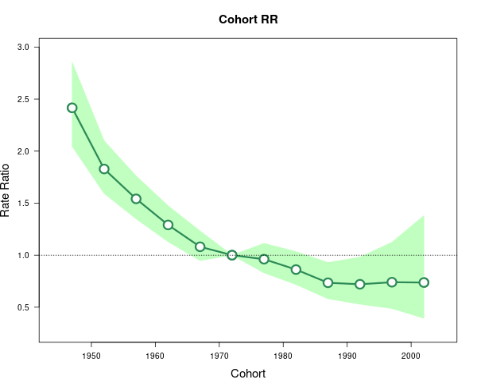**  **I** | |

**Figure S3 Age, period, and cohort trends in global HHD. (A-C) prevalence; (D-F) mortality; (G-I) DALYs.**

| **Location** | **Age(years)** | **Number (95% UI)** | | | **Rate per 100,000 (95% UI)** | | | |  |
| --- | --- | --- | --- | --- | --- | --- | --- | --- | --- |
|  |  | **1990** | **2021** | **percentage**  **change (100%)** | | **1990** | **2021** | **EAPC (95% CI)** | |
| Global | 15-19 | 7,719.73 (4,828.12-11,602.10) | 10,917.65 (6,498.82-17,578.58) | 0.41 | | 3.02 (1.89-4.54) | 3.60 (2.14-5.79) | 0.74 (0.64,0.83) | |
|  | 20-24 | 19,021.28 (12,675.00-27,195.50) | 27,541.17 (17,568.10-41,778.66) | 0.45 | | 7.79 (5.19-11.14) | 9.38 (5.98-14.22) | 0.83 (0.72,0.94) | |
|  | 25-29 | 27,121.00 (17,204.55-37,962.54) | 42,150.42 (25,930.16-63,334.75) | 0.55 | | 12.32 (7.82-17.25) | 14.49 (8.91-21.77) | 0.95 (0.79,1.12) | |
|  | 30-34 | 32,706.84 (19,919.22-48,767.94) | 62,036.53 (37,864.30-93,928.43) | 0.90 | | 17.20 (10.48-25.65) | 20.75 (12.67-31.42) | 1.01 (0.87,1.15) | |
|  | 35-39 | 41,196.41 (25,016.25-61,954.17) | 85,548.71 (49,657.96-133,598.72) | 1.08 | | 23.75 (14.42-35.72) | 30.79 (17.88-48.09) | 0.93 (0.87,0.99) | |
|  | 40-44 | 53,606.16 (33,198.58-82,802.80) | 121,061.72 (73,844.85-193,799.25) | 1.26 | | 38.23 (23.68-59.05) | 48.80 (29.77-78.12) | 0.77 (0.72,0.82) | |
|  | 45-49 | 78,170.69 (52,055.16-112,163.60) | 195,288.69 (126,446.02-285,486.84) | 1.50 | | 68.69 (45.74-98.56) | 82.87 (53.66-121.15) | 0.7 (0.63,0.76) | |
| High SDI | 15-19 | 910.51 (584.54-1,375.95) | 1,109.86 (642.58-1,799.76) | 0.22 | | 2.86 (1.83-4.32) | 3.82 (2.21-6.19) | 1.52 (1.34,1.69) | |
|  | 20-24 | 2,623.49 (1,756.33-1,756.33) | 3,347.84 (2,057.68-5,155.92) | 0.28 | | 7.81 (5.23-11.02) | 10.62 (6.53-16.36) | 1.66 (1.46,1.85) | |
|  | 25-29 | 5,009.97 (3,256.79-7,250.40) | 6,601.99 (4,045.82-9,822.91) | 0.32 | | 13.98 (9.09-20.23) | 19.10 (11.71-28.42) | 1.91 (1.62,2.2) | |
|  | 30-34 | 7,256.11 (4,558.18-10,603.80) | 10,660.12 (6,717.86-15,726.46) | 0.47 | | 20.45 (12.85-29.88) | 28.48 (17.95-42.01) | 1.95 (1.65,2.25) | |
|  | 35-39 | 8,449.29 (5,102.14-12,726.27) | 12,725.01 (7,847.37-18,947.99) | 0.51 | | 25.27 (15.26-38.06) | 33.55 (20.69-49.95) | 1.28 (1.16,1.4) | |
|  | 40-44 | 10,541.18 (6,410.45-16,696.42) | 14,370.17 (9,407.34-21,624.20) | 0.36 | | 33.76 (20.53-53.47) | 39.14 (25.63-58.90) | 0.36 (0.2,0.53) | |
|  | 45-49 | 14,684.70 (9,682.48-21,734.03) | 23,095.15 (15,676.33-8,669.32) | 0.57 | | 58.12 (38.32-86.02) | 64.32 (43.66-91.13) | 0.52 (0.44,0.61) | |
| High-middle SDI | 15-19 | 1,225.57 (769.76-1,880.67) | 1,192.06 (741.43-1,882.46) | -0.03 | | 2.59 (1.63-3.97) | 3.46 (2.15-5.47) | 1.32 (1.18,1.46) | |
|  | 20-24 | 3,122.51 (2,080.45-4,492.21) | 3,228.43 (2,070.64-4,677.26) | 0.03 | | 6.49 (4.32-9.33) | 9.07 (5.82-13.14) | 1.31 (1.23,1.38) | |
|  | 25-29 | 4,353.29 (2,811.62-6,029.78) | 5,397.09 (3,261.23-7,643.29) | 0.24 | | 9.52 (6.15-13.18) | 13.39 (8.09-18.96) | 1.35 (1.21,1.48) | |
|  | 30-34 | 4,672.38 (2,826.79-7,007.01) | 8,253.72 (4,936.22-12,436.22) | 0.77 | | 11.19 (6.77-16.78) | 16.03 (9.59-24.15) | 1.44 (1.29,1.59) | |
|  | 35-39 | 5,454.95 (3,328.28-8,262.18) | 10,787.08 (6,419.26-16,972.56) | 0.98 | | 13.81 (8.43-20.92) | 21.77 (12.95-34.25) | 1.47 (1.31,1.64) | |
|  | 40-44 | 6,894.87 (4,171.39-10,654.14) | 15,873.18 (9,402.94-25,711.55) | 1.30 | | 22.43 (13.57-34.65) | 34.87 (20.66-56.48) | 1.42 (1.24,1.6) | |
|  | 45-49 | 10,713.17 (7,098.41-15,416.86) | 30,364.46 (19,092.27-44,917.34) | 1.83 | | 43.70 (28.95-62.88) | 62.96 (39.59-93.14) | 1.29 (1.16,1.42) | |
| Middle SDI | 15-19 | 2,965.90 (1,877.91-4,526.50) | 3,130.76 (1,896.13-5,117.88) | 0.06 | | 3.22 (2.04-4.91) | 3.56 (2.16-5.83) | 0.45 (0.31,0.59) | |
|  | 20-24 | 7,359.74 (4,857.86-10,502.48) | 8,059.31 (5,126.93-12,165.32) | 0.10 | | 8.33 (5.50-11.89) | 9.30 (5.92-14.04) | 0.6 (0.42,0.78) | |
|  | 25-29 | 9,781.22 (6,239.14-13,775.94) | 12,882.03 (7,694.77-19,015.20) | 0.32 | | 13.06 (8.33-18.39) | 14.22 (8.49-20.99) | 0.76 (0.56,0.96) | |
|  | 30-34 | 10,860.60 (6,482.63-16,160.72) | 20,114.08 (11,874.28-30,412.57) | 0.85 | | 18.09 (10.80-26.92) | 20.37 (12.02-30.80) | 0.76 (0.62,0.9) | |
|  | 35-39 | 14,152.09 (8,774.44-21,091.28) | 29,335.12 (17,000.66-45,688.59) | 1.07 | | 25.53 (15.83-38.05) | 32.01 (18.55-49.85) | 0.73 (0.66,0.81) | |
|  | 40-44 | 18,363.89 (11,169.63-27,901.43) | 43,861.04 (26,474.56-70,222.68) | 1.39 | | 43.46 (26.43-66.03) | 53.59 (32.34-85.79) | 0.63 (0.55,0.7) | |
|  | 45-49 | 26,271.76 (17,696.23-37,325.97) | 73,628.36 (47,181.29-107,787.33) | 1.80 | | 77.51 (52.21-110.13) | 90.77 (58.17-132.89) | 0.56 (0.46,0.65) | |
| Low-middle SDI | 15-19 | 1,846.21 (1,156.14-2,725.04) | 3,281.71 (1,934.66-5,268.18) | 0.78 | | 3.14 (1.97-4.64) | 3.63 (2.14-5.83) | 0.49 (0.44,0.55) | |
|  | 20-24 | 4,263.84 (2,889.90-6,011.83) | 8,186.49 (5,290.87-12,175.67) | 0.92 | | 8.18 (5.54-11.53) | 9.41 (6.08-13.99) | 0.53 (0.46,0.61) | |
|  | 25-29 | 5,883.09 (3,689.32-8,173.00) | 11,424.65 (6,936.56-16,673.90) | 0.94 | | 13.11 (8.22-18.21) | 14.06 (8.54-20.52) | 0.51 (0.41,0.61) | |
|  | 30-34 | 6,997.18 (4,239.43-10,448.12) | 14,887.69 (8,828.04-22,365.60) | 1.13 | | 18.69 (11.32-27.90) | 20.14 (11.94-30.26) | 0.48 (0.42,0.55) | |
|  | 35-39 | 8,927.05 (5,376.00-2,723.27) | 20,864.84 (11,678.50-33,013.53) | 1.34 | | 27.87 (16.78-42.06) | 31.33 (17.54-49.58) | 0.41 (0.38,0.45) | |
|  | 40-44 | 12,302.55 (7,609.64-19,266.46) | 30,436.08 (17,976.34-49,429.88) | 1.47 | | 47.42 (29.33-74.26) | 52.77 (31.16-85.69) | 0.3 (0.27,0.34) | |
|  | 45-49 | 18,383.36 (12,407.54-26,214.76) | 45,215.70 (28,814.50-67,496.46) | 1.46 | | 84.70 (57.16-120.78) | 91.46 (58.29-136.53) | 0.23 (0.22,0.25) | |
| Low SDI | 15-19 | 763.03 (416.22-1,309.79) | 2,192.55 (1,162.11-3,751.77) | 1.87 | | 3.04 (1.66-5.21) | 3.56 (1.88-6.08) | 0.68 (0.6,0.77) | |
|  | 20-24 | 1,631.72 (958.65-2,588.20) | 4,691.94 (2,717.56-7,621.21) | 1.88 | | 7.51 (4.41-11.92) | 8.90 (5.15-14.45) | 0.7 (0.63,0.78) | |
|  | 25-29 | 2,065.60 (1,153.47-3,379.38) | 5,804.11 (3,253.24-9,399.17) | 1.81 | | 11.15 (6.23-18.24) | 13.18 (7.39-21.34) | 0.74 (0.66,0.81) | |
|  | 30-34 | 2,890.23 (1,598.42-4,620.51) | 8,068.62 (4,486.99-13,018.42) | 1.79 | | 19.00 (10.51-30.38) | 21.74 (12.09-35.07) | 0.6 (0.55,0.65) | |
|  | 35-39 | 4,179.89 (2,221.96-6,745.80) | 11,769.64 (6,164.58-19,392.68) | 1.82 | | 32.44 (17.25-52.36) | 36.94 (19.35-60.86) | 0.49 (0.46,0.52) | |
|  | 40-44 | 5,459.06 (3,125.37-8,927.31) | 16,424.68 (9,371.74-27,533.82) | 2.01 | | 55.06 (31.52-90.04) | 62.88 (35.88-105.41) | 0.48 (0.46,0.5) | |
|  | 45-49 | 8,043.53 (5,218.07-11,784.41) | 22,821.22 (14,238.60-34,236.03) | 1.84 | | 96.89 (62.86-141.95) | 109.89 (68.56-164.85) | 0.46 (0.44,0.48) | |

**Table S3 HHD prevalence among WCBA, and age trends, 1990-2021.**

| **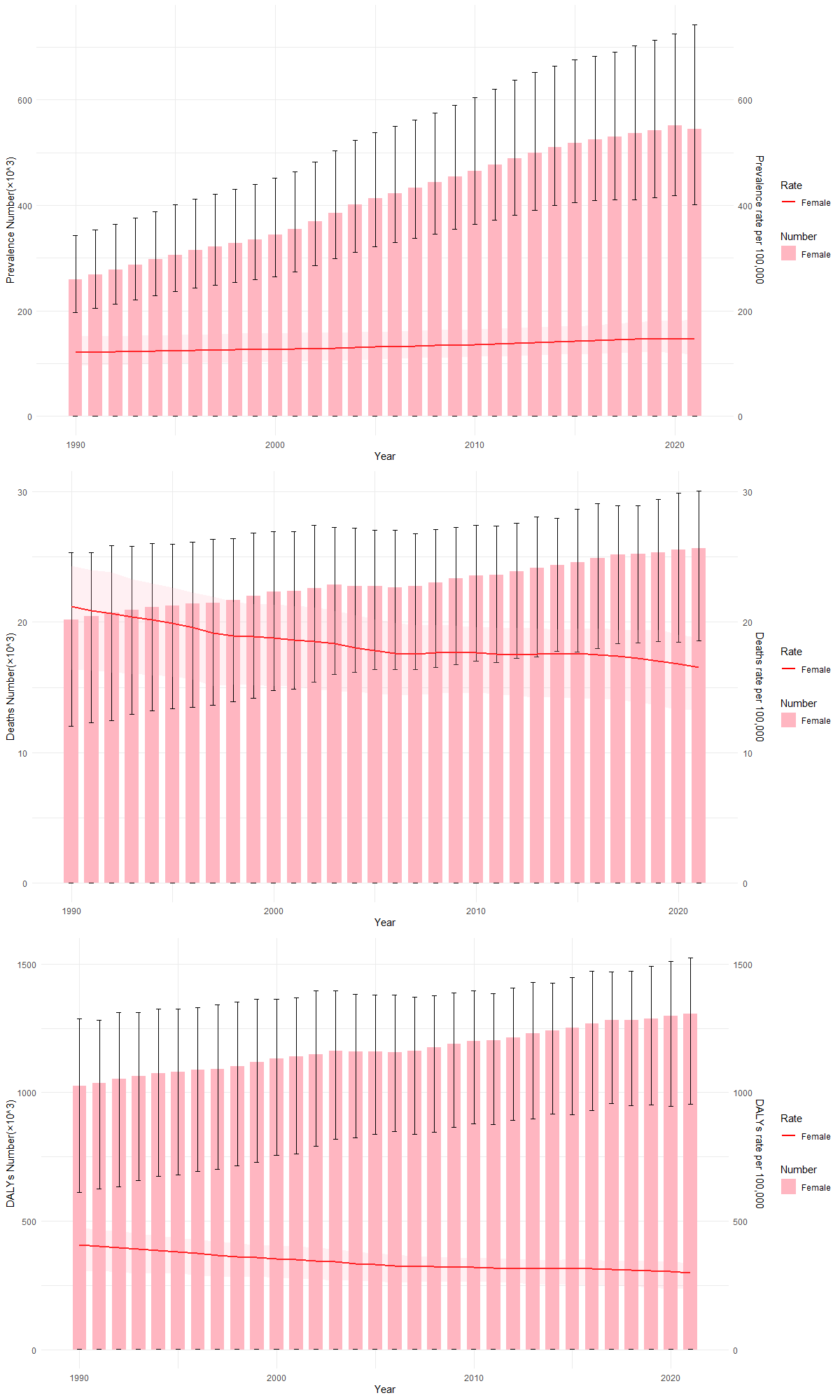**  **Figure S4 Temporal trends in HHD among WCBA from 1990 to 2021.** | **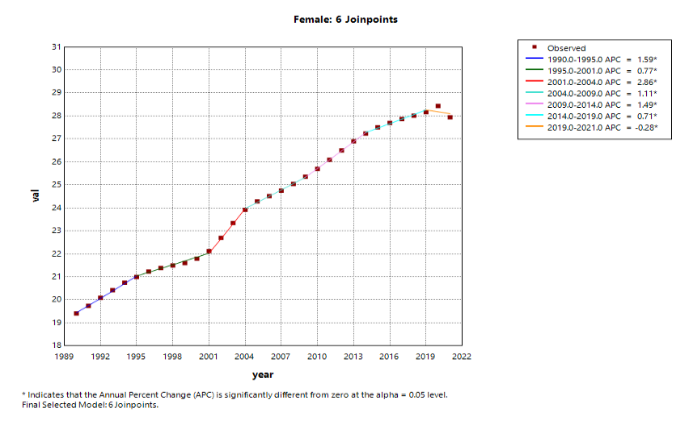A** |
| --- | --- |
|  | **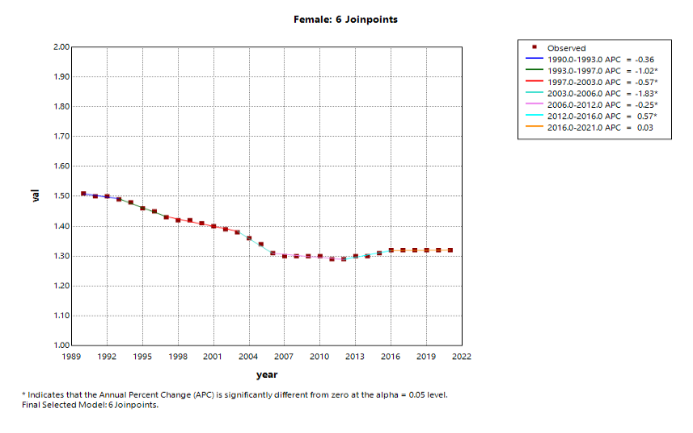B** |
|  | **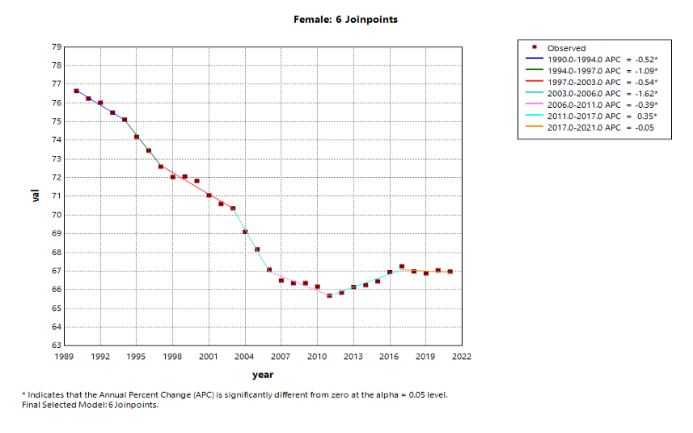 C**  **Figure S5 Joint-point regression analysis of HHD in WCBA.**  **(A) prevalence; (B) mortality; (C) DALYs.** |

| **Prevalence** | | | **Deaths** | | | **DALYs** | | |
| --- | --- | --- | --- | --- | --- | --- | --- | --- |
| **Period** | **APC (95% CI)** | **AAPC (95% CI)** | **Period** | **APC (95% CI)** | **AAPC (95% CI)** | **Period** | **APC (95% CI)** | **AAPC (95% CI)** |
| 1990-1995 | 1.59 (1.54, 1.65) | 1.20 (1.16, 1.23) | 1990-1993 | -0.36 (-0.84, 0.12) | -0.43 (-0.54, -0.32) | 1990-1994 | -0.52 (-0.78, -0.26) | -0.44 (-0.54, -0.33) |
| 1995-2001 | 0.77 (0.71, 0.82) |  | 1993-1997 | -1.02 (-1.46, -0.58) |  | 1994-1997 | -1.09 (-1.86, -0.33) |  |
| 2001-2004 | 2.85 (2.61, 3.11) |  | 1997-2003 | -0.57 (-0.75, -0.39) |  | 1997-2003 | -0.53 (-0.70, -0.37) |  |
| 2004-2009 | 1.11 (1.04, 1.19) |  | 2003-2006 | -1.83 (-2.52, -1.13) |  | 2003-2006 | -1.63 (-2.24, -1.01) |  |
| 2009-2014 | 1.49 (1.41, 1.56) |  | 2006-2012 | -0.25 (-0.40, -0.11) |  | 2006-2011 | -0.39 (-0.57, -0.20) |  |
| 2014-2019 | 0.71 (0.63, 0.79) |  | 2012-2016 | 0.57 (0.24, 0.90) |  | 2011-2017 | 0.35 (0.22, 0.47) |  |
| 2019-2021 | -0.28 (-0.55, -0.02) |  | 2016-2021 | 0.03 (-0.12, 0.17) |  | 2017-2021 | -0.05 (-0.23, 0.12) |  |

**Table S4 Join-point regression analysis of HHD prevalence, mortality and DALYs in WCBA.**

**
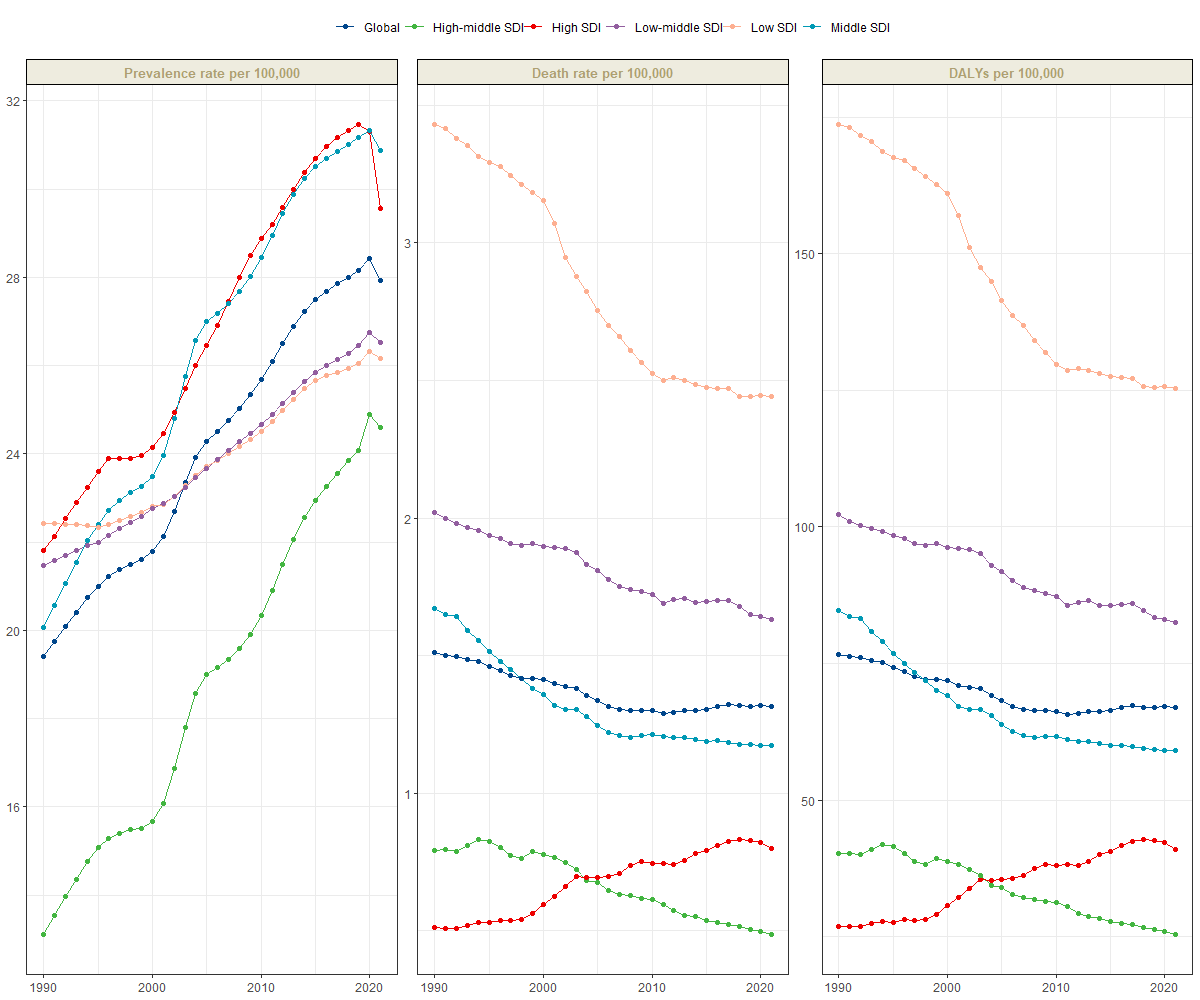
**

**Figure S6 Trends in HHD prevalence, mortality, and DALYs rates among WCBA by SDI, 1990-2021.**

| **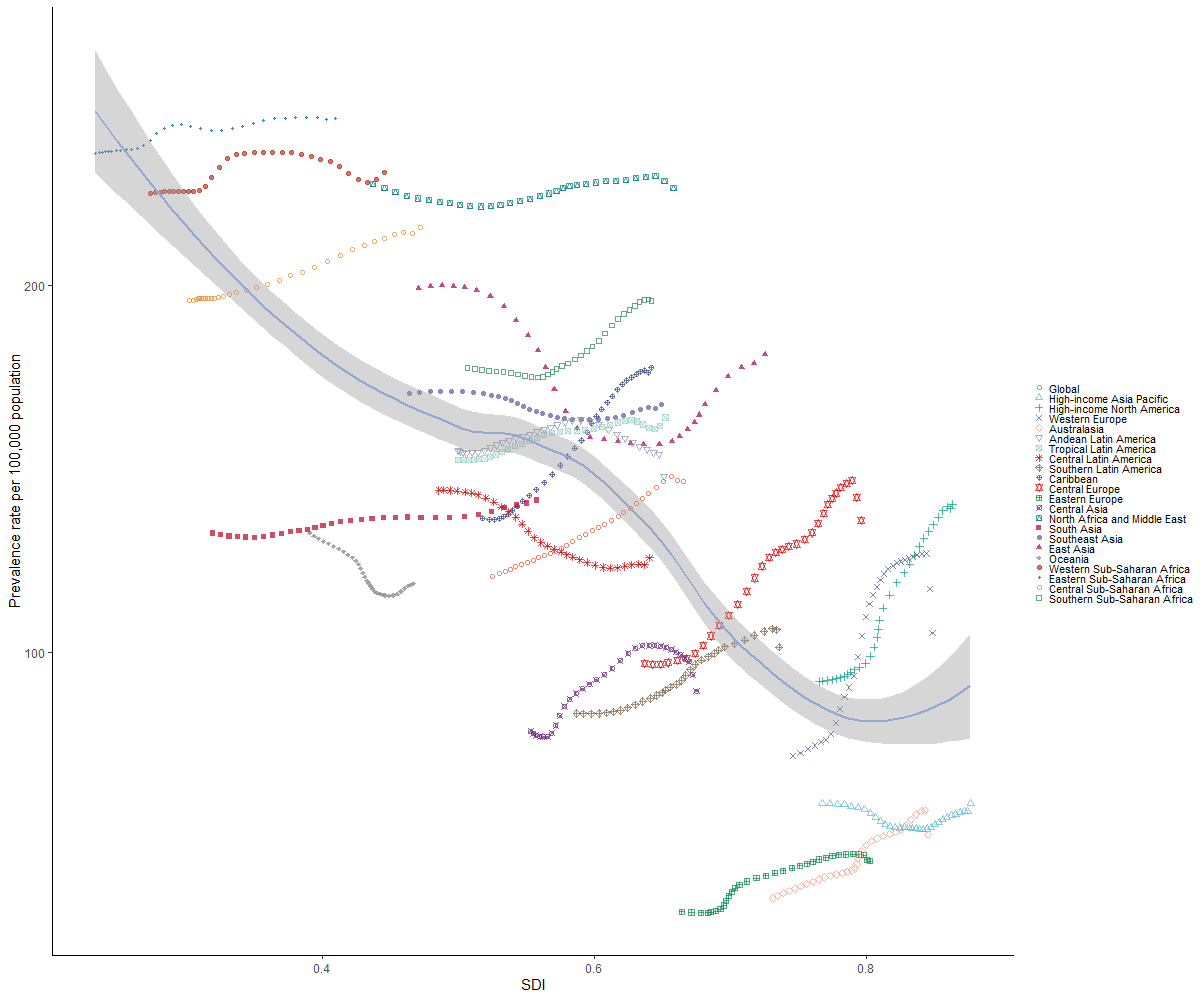A** |
| --- |
| **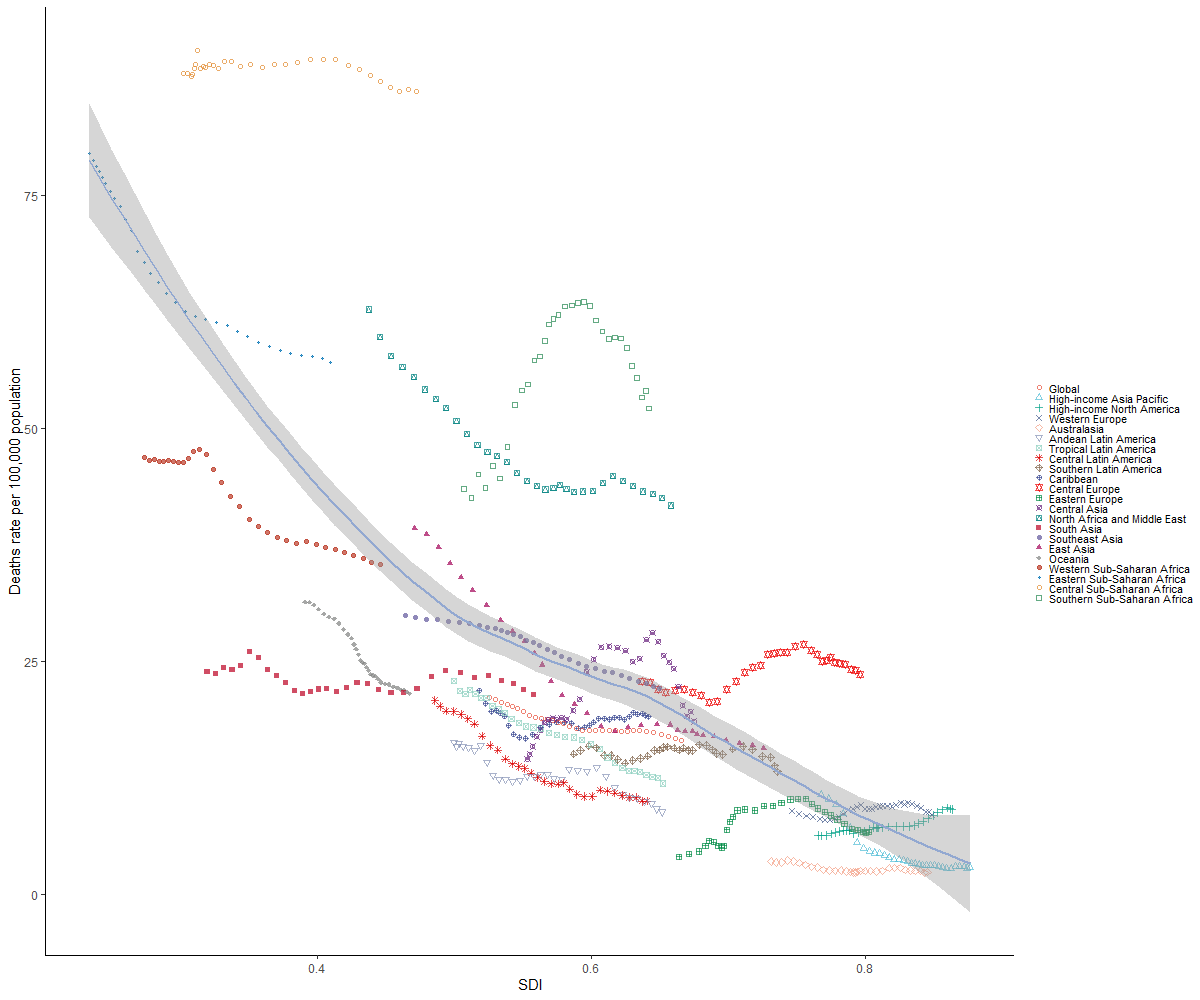B** |
| **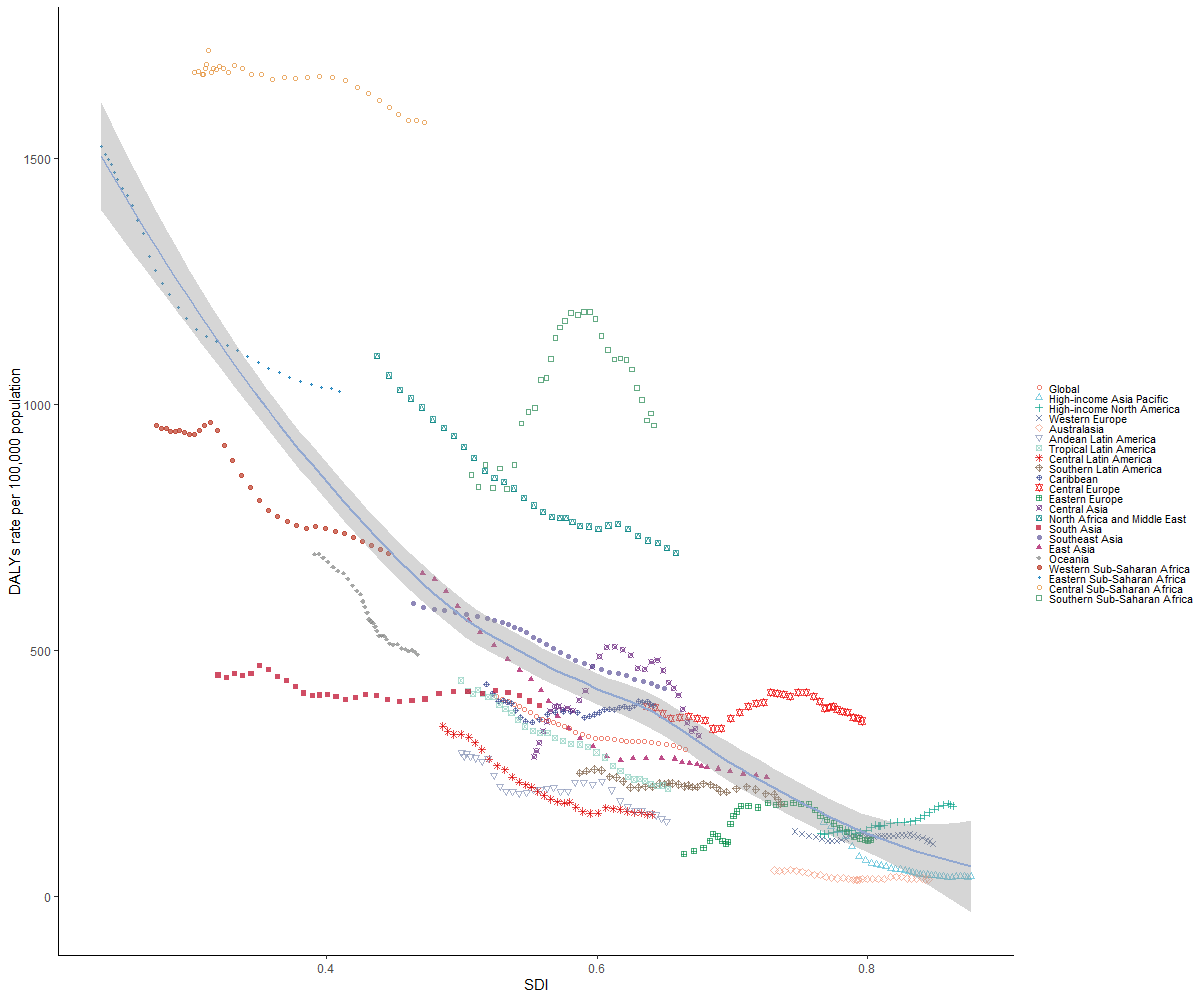C** |
| **Figure S7 Relationship between SDI and HHD prevalence, mortality, and DALYs WCBA in 21 GBD regions.** **Expected values based on Socio-demographic Index and disease rates in all locations are shown as the blue line with grey area. (A) prevalence; (B) mortality (C) DALYs.** |

| **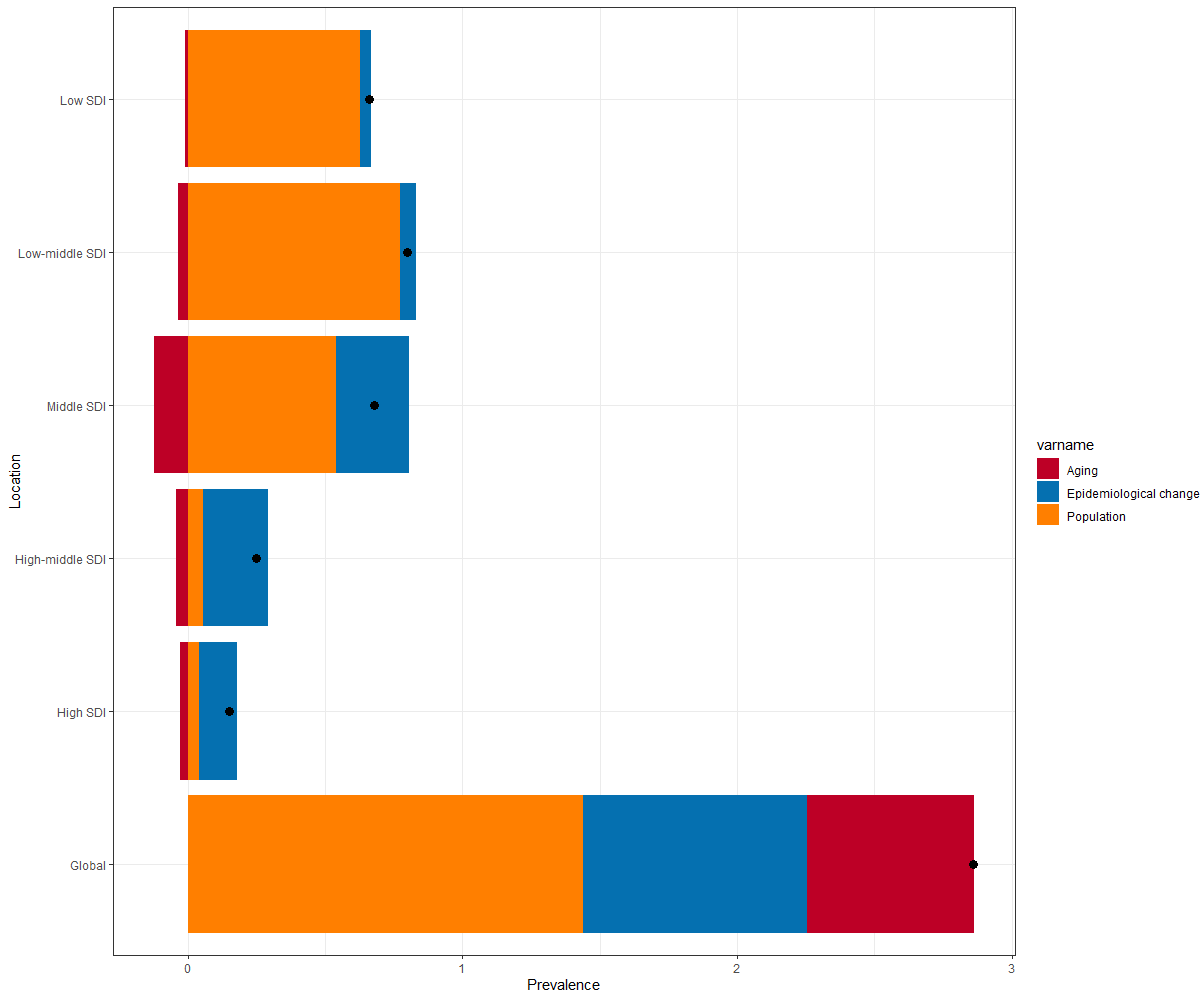A** |
| --- |
| **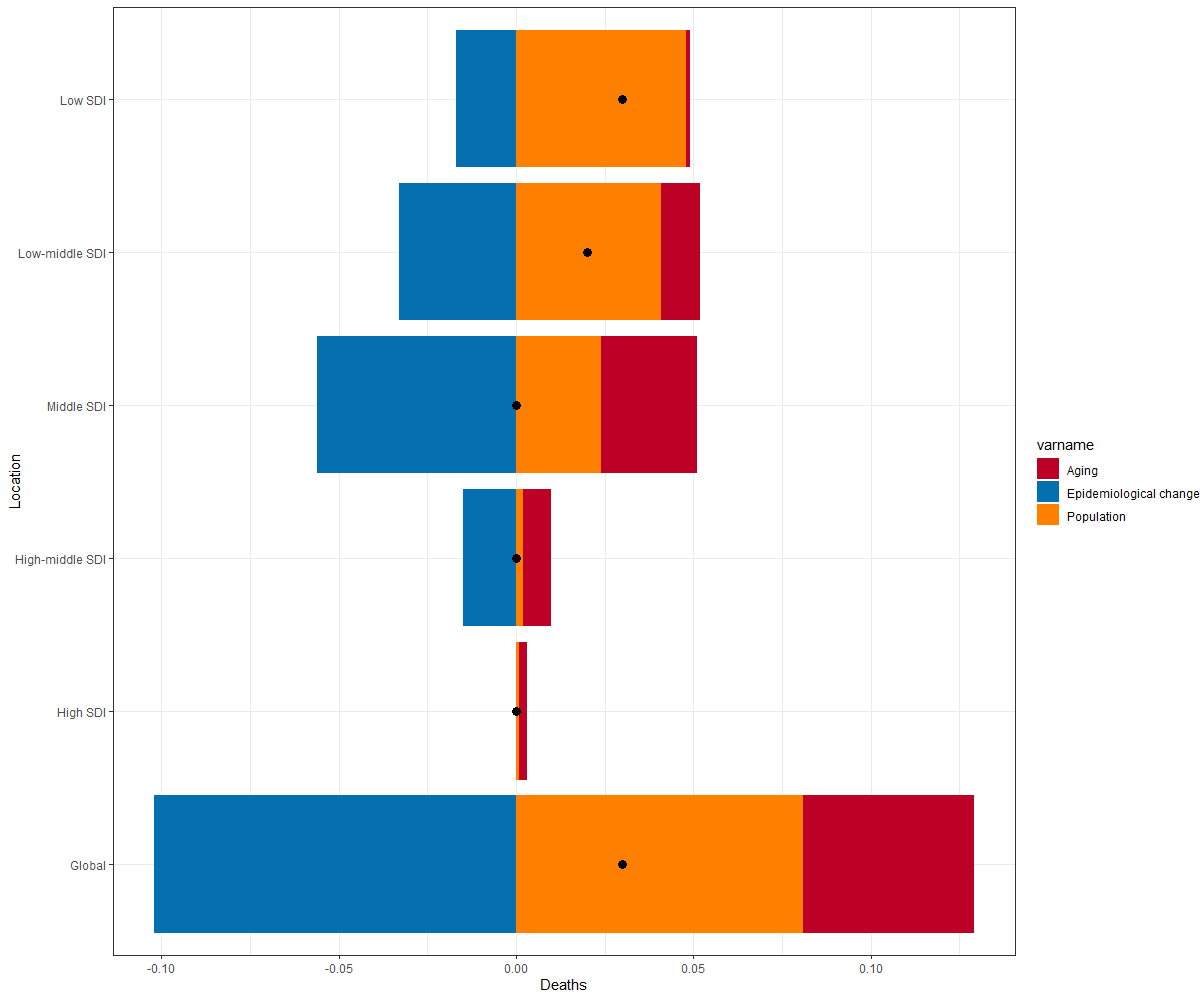B** |
| **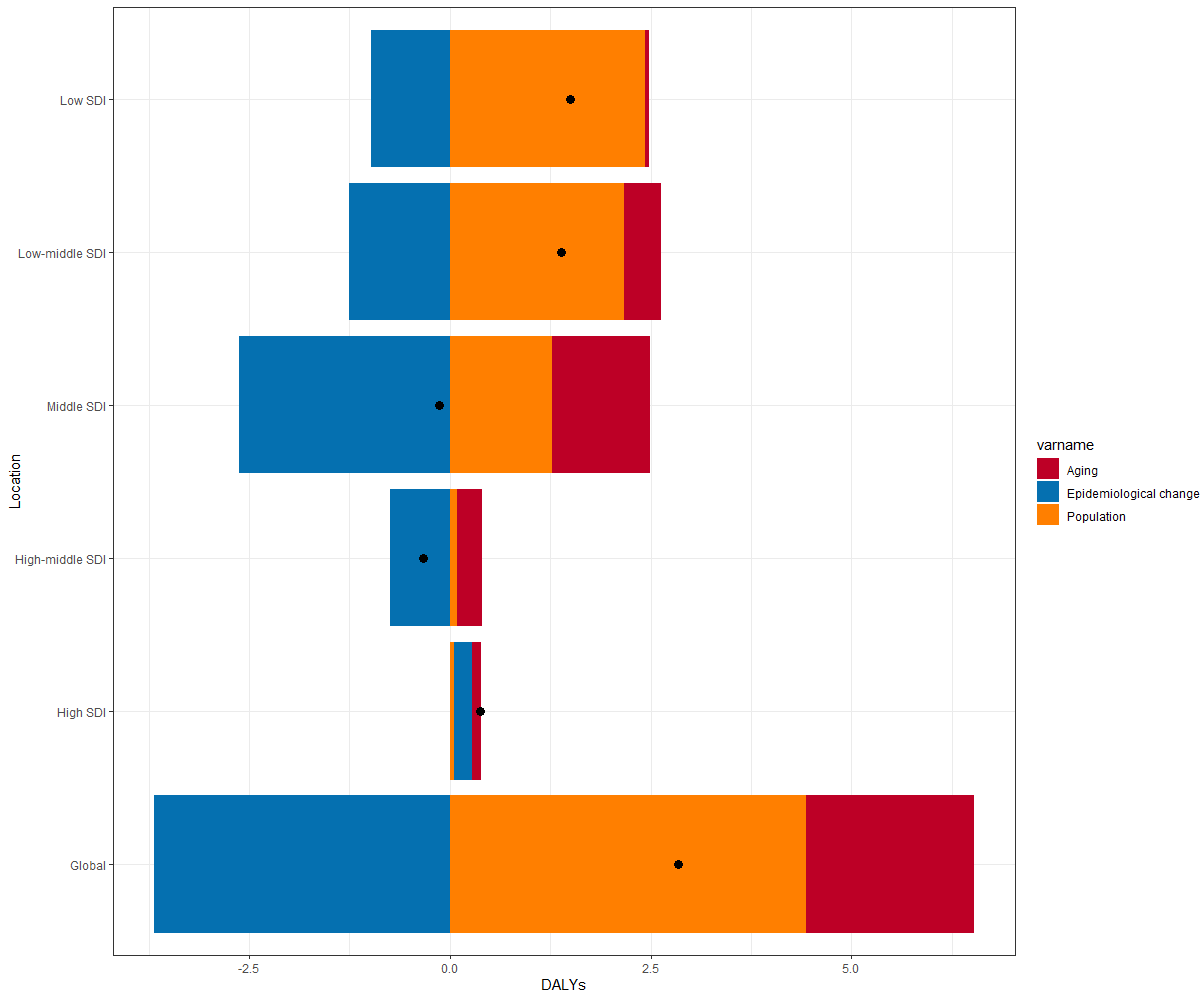C** |
| **Figure S8 Changes in HHD DALYs according to population-level determinants of population growth, aging, and epidemiological change from 1990 to 2021 at the global level and by SDI quintile. The black dot represents the overall value of change contributed by all 3 components. For each component, the magnitude of a positive value indicates a corresponding increase in HHD DALYs attributed to the component; the magnitude of a negative value indicates a corresponding decrease in HHD DALYs attributed to the related component. (A) prevalence; (B) mortality; (C) DALYs** |

**
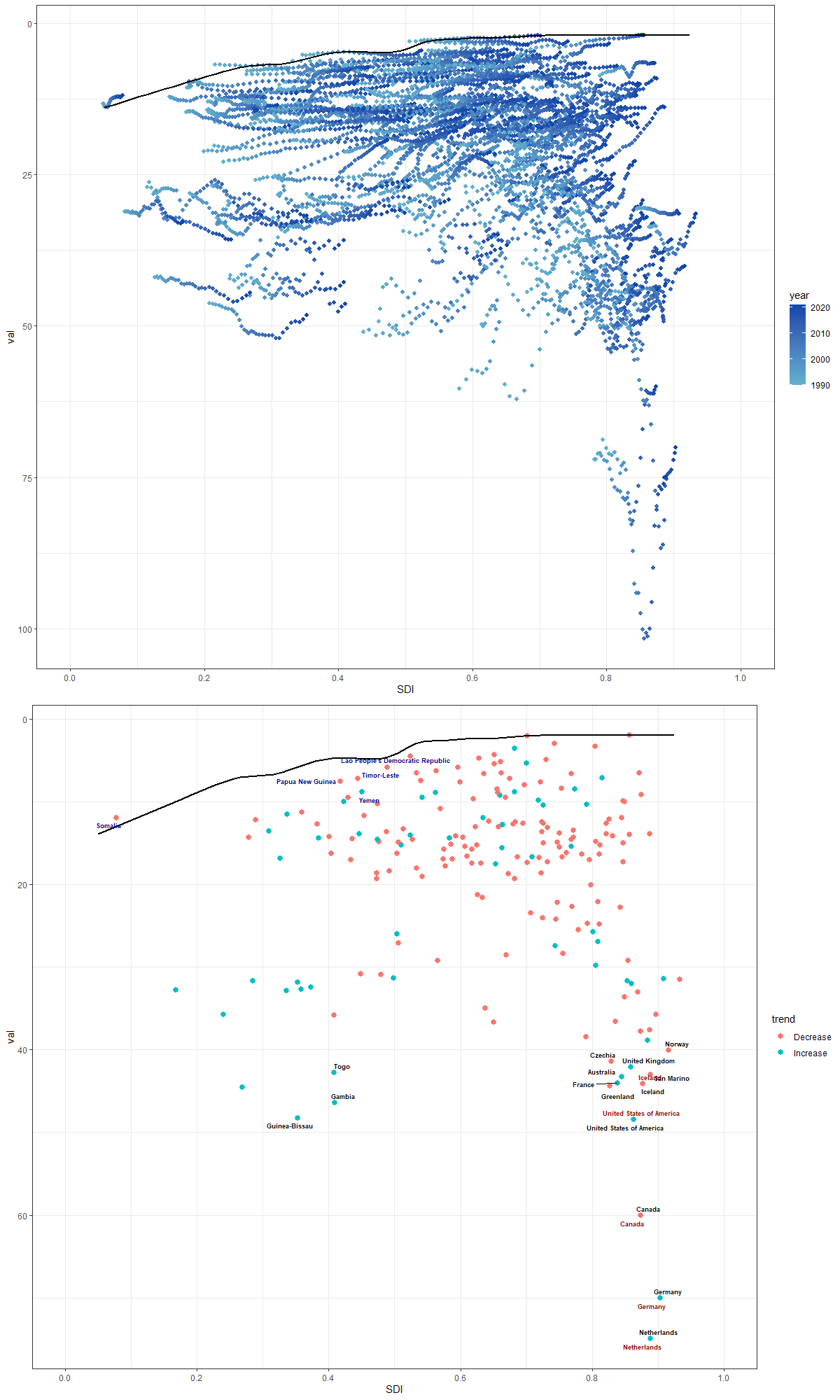
**

**Figure S9 Frontier analysis based on SDI and DALYs rate of HHD among WCBA in 2021. The frontier is delineated in solid black color; countries and territories are represented as dots. The top 15 countries with the largest effective difference are labeled in black; examples of frontier countries with low SDI (< 0.5) and low effective difference are labeled in blue, and examples of countries and territories with high SDI (> 0.85) and relatively high effective difference for their level of development are labeled in red. Red dots indicate an increase in DALYs rate of HHD among WCBA from 1990 to 2021; blue dots indicate a decrease in DALYs rate of HHD among WCBA from 1990 to 2021.**
